# The Morphogenetic Protein CotE Positions Exosporium Proteins CotY and ExsY during Sporulation of Bacillus cereus

**DOI:** 10.1128/mSphere.00007-21

**Published:** 2021-04-21

**Authors:** Armand Lablaine, Mònica Serrano, Christelle Bressuire-Isoard, Stéphanie Chamot, Isabelle Bornard, Frédéric Carlin, Adriano O. Henriques, Véronique Broussolle

**Affiliations:** a INRAE, Avignon Université, UMR SQPOV, Avignon, France; b Instituto de Tecnologia Quimica e Biologica, Universidade Nova de Lisboa, Oeiras, Portugal; c INRAE, Pathologie végétale, Montfavet, France; University of Iowa

**Keywords:** spore, morphogenetic proteins, exosporium, SR-SIM, endospores

## Abstract

The exosporium is the outermost spore layer of some *Bacillus* and *Clostridium* species and related organisms. It mediates the interactions of spores with their environment, modulates spore adhesion and germination, and has been implicated in pathogenesis. In *Bacillus cereus*, the exosporium consists of a crystalline basal layer, formed mainly by the two cysteine-rich proteins CotY and ExsY, surrounded by a hairy nap composed of glycoproteins. The morphogenetic protein CotE is necessary for the integrity of the B. cereus exosporium, but how CotE directs exosporium assembly remains unknown. Here, we used super-resolution fluorescence microscopy to follow the localization of SNAP-tagged CotE, CotY, and ExsY during B. cereus sporulation and evidenced the interdependencies among these proteins. Complexes of CotE, CotY, and ExsY are present at all sporulation stages, and the three proteins follow similar localization patterns during endospore formation that are reminiscent of the localization pattern of Bacillus subtilis CotE. We show that B. cereus CotE guides the formation of one cap at both forespore poles by positioning CotY and then guides forespore encasement by ExsY, thereby promoting exosporium elongation. By these two actions, CotE ensures the formation of a complete exosporium. Importantly, we demonstrate that the assembly of the exosporium is not a unidirectional process, as previously proposed, but occurs through the formation of two caps, as observed during B. subtilis coat morphogenesis, suggesting that a general principle governs the assembly of the spore surface layers of *Bacillaceae*.

**IMPORTANCE** Spores of *Bacillaceae* are enveloped in an outermost glycoprotein layer. In the B. cereus group, encompassing the Bacillus anthracis and B. cereus pathogens, this layer is easily recognizable by a characteristic balloon-like appearance and separation from the underlying coat by an interspace. In spite of its importance for the environmental interactions of spores, including those with host cells, the mechanism of assembly of the exosporium is poorly understood. We used super-resolution fluorescence microscopy to directly visualize the formation of the exosporium during the sporulation of B. cereus, and we studied the localization and interdependencies of proteins essential for exosporium morphogenesis. We discovered that these proteins form a morphogenetic scaffold before a complete exosporium or coat is detectable. We describe how the different proteins localize to the scaffold and how they subsequently assemble around the spore, and we present a model for the assembly of the exosporium.

## INTRODUCTION

Bacterial endospores are one of the most resistant life forms (1). Endospores (here referred to as “spores” for simplicity) formed by members of the *Firmicutes* share a general morphological plan and are composed of several concentric layers. The core is the most internal structure and contains the bacterial chromosome. The core is surrounded by an “inner” membrane, then by a thin layer of peptidoglycan (the germ cell wall), and then by an external and thicker layer of modified peptidoglycan (the cortex). The cortex, delimited by the outer forespore membrane (OFM), is enveloped in two main proteinaceous layers: the coat and the exosporium. While the coat is common to all species of spore-forming bacteria, the exosporium is found only in some *Bacillus* and *Clostridium* species and related organisms. In the Bacillus cereus group, encompassing the foodborne pathogen B. cereus sensu stricto, the etiologic agent of anthrax, Bacillus anthracis, and the entomopathogenic species Bacillus thuringiensis, the exosporium appears under transmission electron microscopy (TEM) as an irregular balloon-like structure, separated from the rest of the spore by the interspace, an electron-transparent region of unknown composition. The exosporium is described as a thin “basal layer” with a paracrystalline structure surrounded by a “hairy nap” composed of different glycoproteins (2, 3). Whether the exosporium is somehow directly linked to the coat across the interspace remains unclear (4).

The exosporium directly contacts the environment, including host cells and the host immune system. It has a role in adhesion to abiotic surfaces and contributes to protection against predation and internalization by macrophages, as shown for B. anthracis spores (5). Despite its importance, assembly of the exosporium remains a poorly understood process. Electron microscopy reveals that its formation begins early in sporulation, at the onset of engulfment of the forespore by the mother cell, when the engulfing membranes start to curve, and before the coat or the cortex becomes recognizable (4, 6, 7). At this stage, the exosporium forms a single electron-dense structure, named the “cap,” adjacent to the OFM at the mother cell-proximal (MCP) forespore pole (4, 6, 7). After the completion of engulfment, the cap appears more markedly electron dense and becomes separated from the OFM by the interspace (4, 6, 7). The noncap part of the exosporium is detected later, concomitantly with coat deposition (7). The assembly of the coat and the assembly of the exosporium appear to be interdependent, as shown recently for B. anthracis, where the formation of the cap part of the exosporium interferes with coat deposition at this site (7). Indeed, the coat assembles first along the longitudinal sides of the forespore, then at the mother cell-distal (MCD) forespore pole, and finally at the MCP forespore pole (7).

The exosporium is composed of at least 20 different proteins (5). Among them, ExsY, the main structural component of the exosporium basal layer, self-assembles into large 2-dimensional crystalline arrays (8). B. cereus *exsY* mutant spores are devoid of an exosporium, but a cap-like structure is observed during the sporulation of a B. anthracis *exsY* mutant on agar plates (9, 10). Strikingly, CotY self-assembles like its paralogue ExsY and may form the basal layer of the MCP cap structure observed in B. anthracis *exsY* mutant spores (8, 10). Both ExsY and CotY are cysteine-rich proteins, and cooperative disulfide bond formation seems to play a role in their self-assembly (8). The current model of exosporium assembly proposes that CotY forms the cap, possibly in association with ExsY (5, 8). From this cap, ExsY self-polymerizes to assemble unidirectionally towards the MCD pole, forming the remaining noncap part of the basal layer. This structure offers a scaffold for the assembly of additional exosporium proteins, such as BxpB, required for the attachment of the collagen-like glycoproteins that form the hairy nap (11–17).

ExsY and CotY are orthologues of Bacillus subtilis CotZ and CotY, which are important structural crust components (9, 18–20), and the self-organization of these cysteine-rich proteins plays an important role in the formation of both layers (8, 21). Moreover, the morphogenetic protein CotE guides both outer coat and crust assembly in B. subtilis (18, 19, 22), and its B. cereus CotE orthologue is required for exosporium assembly (6, 23). These observations suggest that the assembly mechanisms of the crust and exosporium could be similar. The assembly of the proteins forming the different layers of the B. subtilis coat is a two-step process. First, a group of early-synthesized proteins, mainly morphogenetic proteins, forms an organizational center on the MCP forespore pole, named the cap, which is dependent on the morphogenetic ATPase SpoIVA (20, 24). CotE and CotZ are part of this cap (20). In the second step, the different coat proteins encapsulate the circumference of the spore from the single cap, a process that requires SpoVID and is named encasement (25). Encasement by CotE depends on a direct interaction with SpoVID and involves the formation of a second cap at the MCD following engulfment completion, at the site of membrane fission (20, 25, 26). Other proteins, designated kinetic class II proteins (20), share this sequence of localization and encasement.

How the exosporium proteins localize during the course of sporulation, which proteins dictate the different steps of exosporium assembly, and on which interactions the process relies are unknown for the B. cereus group. In particular, how CotE contributes to the assembly of the exosporium is unknown. Here, we study the localization and the dependencies between CotY, ExsY, and CotE during B. cereus sporulation using super-resolution fluorescence microscopy. We show that CotY, ExsY, and CotE exhibit the same pattern of assembly throughout sporulation, a pattern reminiscent of the localization of the kinetic class II coat proteins of B. subtilis to which CotE belongs. Importantly, our results also reveal that CotE forms complexes with CotY and ExsY during sporulation and that CotE interacts directly with these proteins. CotE guides the assembly of CotY and ExsY via direct and indirect interactions, allowing the formation of two cap structures and the subsequent elongation of the morphogenetic scaffold of the exosporium. These two crucial steps ensure the progressive assembly of the exosporium around the spore. These features are highly similar to those observed during coat layer formation in B. subtilis, suggesting that the principle governing the assembly of spore surface layers is conserved among distant members of the *Bacillus* group.

## RESULTS

### CotY, ExsY, and CotE follow the same assembly dynamics during sporulation.

To determine the role of CotE, together with CotY and ExsY, in the formation of the B. cereus exosporium, we first monitored the localization of these three proteins during sporulation by using super-resolution structured illumination microscopy (SR-SIM) (27). We used translational fusions to the SNAP tag, which becomes fluorescent (red signal on overlay images) upon covalent binding of the TMR-Star substrate (28–31). The SNAP fusions were constructed using a low-copy-number plasmid, which we used previously to complement B. cereus *cotE* mutant cells (23). However, spores of the complemented *cotE* mutant showed partial exosporium attachment (23), and we therefore preferred to study the localization of SNAP proteins in a wild-type (WT) background. We checked by conventional fluorescence microscopy that the localization patterns of CotE-SNAP (SNAP at the C-terminal end of CotE) and SNAP-CotE (SNAP at the N-terminal end of CotE) in *cotE* mutant and WT cells harboring those constructions were identical, i.e., without any bias caused by additional copies of *cotE* (data not shown). Previous work also showed that more B. cereus CotE was extracted in spores formed at 20°C than at 37°C (23). We therefore performed sporulation of ATCC 14579 cells producing CotY-, ExsY-, or CotE-SNAP fusions or SNAP-CotE at 20°C and showed that their patterns of localization during sporulation at 20°C and 37°C were identical (data not shown). Culture samples were collected throughout sporulation and were labeled with TMR, and the different patterns of SNAP fusion localization were scored with respect to the stages of sporulation identified using the MitoTracker Green (MTG) dye (green fluorescent signal on overlay images) (32).

**FIG 1:**
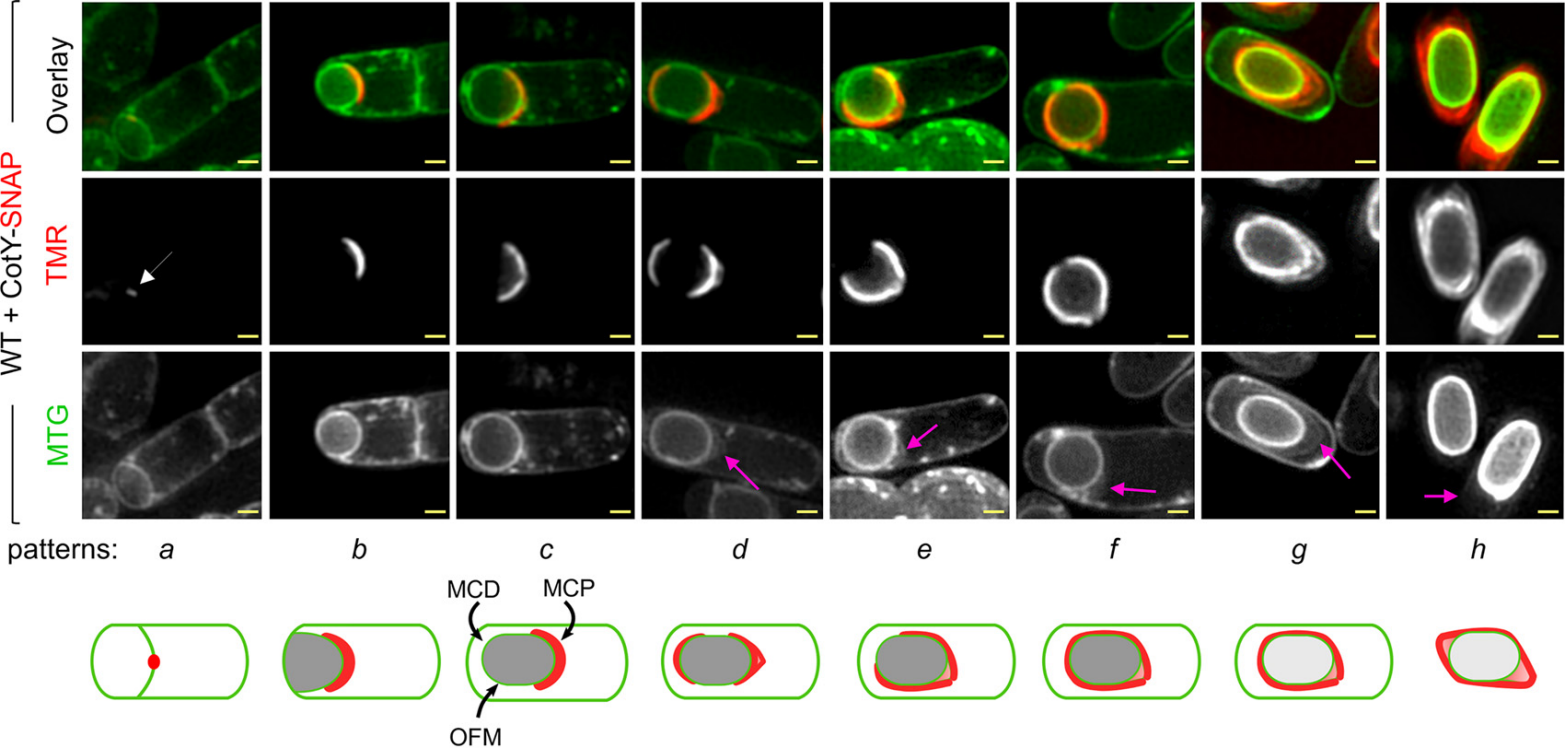
Stages of CotY-SNAP localization during sporulation. Sporulating cells of B. cereus producing CotY-SNAP were labeled with the SNAP substrate TMR-Star and with the membrane dye MTG and were imaged by SR-SIM. Images of a representative cell for each pattern of localization identified at a particular stage of sporulation are shown, with schematic representations (patterns *a* to *h*) below. The white arrow points to the weak signal of CotY-SNAP detected at curved septa; pink arrows point to the exosporium at different stages of its formation; curved black arrows point to the mother cell-proximal (MCP) and -distal (MCD) forespore poles and to the outer forespore membranes (OFM). Bars, 0.5 μm. The stages of ExsY-SNAP, SNAP-CotE, and CotE-SNAP localization during sporulation are shown in Fig. S1A, Fig. S1B, and Fig. S2A, respectively.

We first detected CotY-SNAP as a discrete fluorescent signal on curved septa at the onset of engulfment (Fig. 1, pattern *a*, white arrow) in 13% and 2% of the sporangia scored after hour 20 and hour 24 of sporulation, respectively (see Fig. S1C in the supplemental material). This signal then extended along the engulfing membranes and became cap-shaped, representing 33% and 7% of the sporangia after hour 20 and hour 24 of sporulation, respectively (Fig. 1 and Fig. S1C, pattern *b*). Importantly, the cap pattern of CotY-SNAP persisted until the end of engulfment, representing 33% and 25% of the sporangia at hours 20 and 24 of sporulation, respectively (Fig. 1 and Fig. S1C, pattern *c*). At hour 24, we distinguished different patterns of CotY-SNAP localization in sporangia that had completed engulfment: (i) a red fluorescent cap-shaped signal at the MCP pole in 25% of the sporangia (Fig. 1 and Fig. S1C, pattern *c*), (ii) caps at both forespore poles in 10% of the sporangia (pattern *d*), (iii) a red fluorescent signal covering three-quarters of the forespore in 26% of the sporangia (pattern *e*), and (iv) a ring of red fluorescence in 30% of the sporangia (pattern *f*). Remarkably, in fully engulfed sporangia, a layer labeled by MTG was detected at the MCP forespore pole, separated from the forespore membrane (Fig. 1, pink arrows, patterns *d*, *e*, and *f*). Strikingly, the CotY-SNAP signal appeared to migrate from the OFM (Fig. 1, pattern *c*) to this nascent layer (patterns *d*, *e*, and *f*). Later, this layer exhibited the recognizable balloon-like structure of the exosporium and completely surrounded the spore, showing that MTG also labeled the exosporium (Fig. 1, pink arrows, patterns *g* and *h*). Hence, this layer, when detected at the MCP forespore pole in sporangia that had completed engulfment, likely corresponds to the MCP cap of the exosporium (patterns *d*, *e*, and *f*). From hour 48, only sporangia of phase-bright spores and free spores were observed by phase-contrast microscopy (data not shown), and the CotY-SNAP signal was clearly superimposed on the exosporium basal layer (Fig. 1, pattern *g*). In free spores, the signal was present in the different regions of the exosporium (Fig. 1, pattern *h*). Similar localization patterns were observed for ExsY-SNAP and SNAP-CotE (Fig. S1A, B, and C). The dynamic of CotE-SNAP assembly was globally similar, with slight differences in sporangia that had completed the engulfment process and presented a transient defect in MCP cap separation from the OFM (Fig. S2 and Text S1).

10.1128/mSphere.00007-21.1 FIG S1 Stages of ExsY-SNAP and SNAP-CotE localization during sporulation. (A and B) Sporulating cells of B. cereus producing ExsY-SNAP (A) or SNAP-CotE (B) were labeled with the SNAP substrate TMR-Star and with the membrane dye MTG and imaged by SR-SIM. Cells representative of the different localization patterns seen (*a* to *h*) (see Fig. 1) are presented. TMR panels correspond to the signal from the SNAP fusion; MTG corresponds to the membrane signal. Pink arrows point to the exosporium at the different steps of its formation. Scale bar, 0.5 μm. (C) The percentage of sporulating cells showing the indicated patterns at the different times of sampling is presented for CotY-SNAP, ExsY-SNAP, and SNAP-CotE. Download FIG S1, TIF file, 0.3 MB. Copyright © 2021 Lablaine et al. 2021 Lablaine et al. https://creativecommons.org/licenses/by/4.0/ This content is distributed under the terms of the Creative Commons Attribution 4.0 International license.

10.1128/mSphere.00007-21.2 FIG S2 Fusion of the SNAP tag to the C terminus of CotE affects the separation of the MCP cap after engulfment completion. (A) Sporulating WT cells of B. cereus producing SNAP-CotE were labeled with TMR-Star and MTG and imaged by SR-SIM. The blue arrow points to a layer formed by CotE-SNAP distinct from the exosporium layer (pink arrows). This layer was not observed with the other SNAP fusions tested (as shown in panels A and B; also Fig. 1). Scale bar, 0.5 μm. Thin sections of a representative CotE-SNAP-producing sporulating cell (B) after engulfment completion, showing a prominent cap at the MCP forespore pole (black arrow). Scale bar, 200 nm; (C) following coat formation, the exosporium appeared to have a normal structure. Scale bar, 500 nm. Ex, exosporium; Ct, coat. Download FIG S2, TIF file, 0.3 MB. Copyright © 2021 Lablaine et al. 2021 Lablaine et al. https://creativecommons.org/licenses/by/4.0/ This content is distributed under the terms of the Creative Commons Attribution 4.0 International license.

10.1128/mSphere.00007-21.9 TEXT S1 Supplemental results and discussion. Download Text S1, DOCX file, 0.04 MB. Copyright © 2021 Lablaine et al. 2021 Lablaine et al. https://creativecommons.org/licenses/by/4.0/ This content is distributed under the terms of the Creative Commons Attribution 4.0 International license.

Taken together, these results show that CotY, ExsY, and CotE share a common pattern of localization during sporulation, behaving as cap proteins at the MCP forespore pole during engulfment; then, after engulfment completion, localizing as a second cap at the MCD spore pole; and finally completely encasing the forespore. Strikingly, during encasement by CotE, CotY, and ExsY, the exosporium MCP cap structure (Fig. 1 and Fig. S1 and S3, patterns *e* and *f*, pink arrows) can be differently oriented with regard to the mother cell (MC) axis. This suggests that the MCP cap becomes mobile after its separation from the OFM (Fig. S3 and Text S1). Moreover, the detection of an off-center MCP exosporium cap appears linked to the observation of a three-quarters-of-a-circle localization with an MCD pole not encased by CotY-SNAP, ExsY-SNAP, or SNAP-CotE (Fig. S1, pattern *e*, and Fig. S3). Indeed, cells expressing CotE-SNAP, which presents a defect in MCP cap separation, always presented a three-quarters-of-a-circle localization with an encased MCD pole and showed a small fluorescence gap only at one longitudinal forespore side (Fig. S3E). Those results show that encasement by CotE, but also by CotY and ExsY, starts at the MCD pole and occurs asymmetrically, covering the rest of the forespore one side before the other (Fig. S3 and Text S1). Finally, we found that encasement of the forespore by CotY, ExsY, and CotE is completed early in most of the sporangia, i.e., from hour 24 (Fig. 1 and Fig. S1, pattern *f*). Importantly, at the same time, coat was not visible on TEM images, and only the cap region of the exosporium was visible on TEM or SR-SIM images (Fig. 1 and Fig. S1 and S4B). Hence, the localization of CotE, CotY, and ExsY does not give rise to any structure visible by TEM in the noncap region, in contrast to the MCP region. Thus, those proteins are present, before coat detection, in the noncap region as an immature morphogenetic scaffold.

10.1128/mSphere.00007-21.3 FIG S3 Asymmetric bidirectional encasement by proteins of the exosporium morphogenetic scaffold. SR-SIM images of sporulating WT cells producing CotY-SNAP (A to C) or CotE-SNAP (D and E) show pattern *d* (C and D) or pattern *e* (A, B and E). Sporulating cells with an exosporium MCP cap, not orientated in the mother cell (MC) axis, present a three-quarters-of-a-circle localization pattern of CotY-SNAP (A and B) with an MCD forespore not encased (A) or an MCD pole encased or partly encased by CotY-SNAP (B). The yellow arrows represent the MC axis, and the pink arrows show the orientation of the exosporium MCP cap structure. (C) Sporulating cells with an exosporium cap structure in the MC axis show a two-caps localization of CotY-SNAP. The blue arrows indicate that the exosporium cap is in the MC axis. CotE-SNAP sporangia present a connected MCP exosporium in the MC axis. (D) First, the cells are not covered by CotE-SNAP on the forespore longitudinal side. (E) Before complete encasement by CotE-SNAP, cells present only a gap of fluorescence on one longitudinal side of the forespore, showing asymmetric encasement (orange arrows). A scheme represents the different orientations of exosporium cap structures, relative to the MC axis, the relative pattern of SNAP-protein localization (red line), and the number of sporangia scored in each category. Only images of the most abundant patterns are presented. Scale bars, 0.5 μm. Download FIG S3, TIF file, 0.5 MB. Copyright © 2021 Lablaine et al. 2021 Lablaine et al. https://creativecommons.org/licenses/by/4.0/ This content is distributed under the terms of the Creative Commons Attribution 4.0 International license.

10.1128/mSphere.00007-21.4 FIG S4 The absence of CotE impairs cap formation and leads to a modified pattern of coat deposition. Thin sections of sporulating B. cereus ATCC 14579 (A to D) or *cotE* (E to H) cells, collected after hour 24 (A and B), hour 28 (C and D), hour 31 (E), or hour 48 (F to H) of sporulation at 20°C, were observed by TEM. (A) A forespore presenting a cap at the MCP; (B) a cap separated from the forespore surface; (C) coat deposition on the longitudinal side; (D) coat deposition on the longitudinal sides and at the MCD forespore pole. (E to H) Forespore of a *cotE* mutant after engulfment completion (E), without any visible coat or cortex (F), with a visible cortex and without any cap or exosporium material, in contrast with the WT at a similar stage of development (A to C). (G and H) A *cotE* forespore with altered coat assembly (G) or with a large accumulation of exosporium material in the mother cell cytoplasm (H). FM, forespore membranes; Ex, exosporium material (cap in A, B, C); Cx, cortex; Ct, coat. Scale bar, 200 nm. Download FIG S4, TIF file, 0.5 MB. Copyright © 2021 Lablaine et al. 2021 Lablaine et al. https://creativecommons.org/licenses/by/4.0/ This content is distributed under the terms of the Creative Commons Attribution 4.0 International license.

### CotE is required for the localization of CotY and ExsY as two caps.

**FIG 2:**
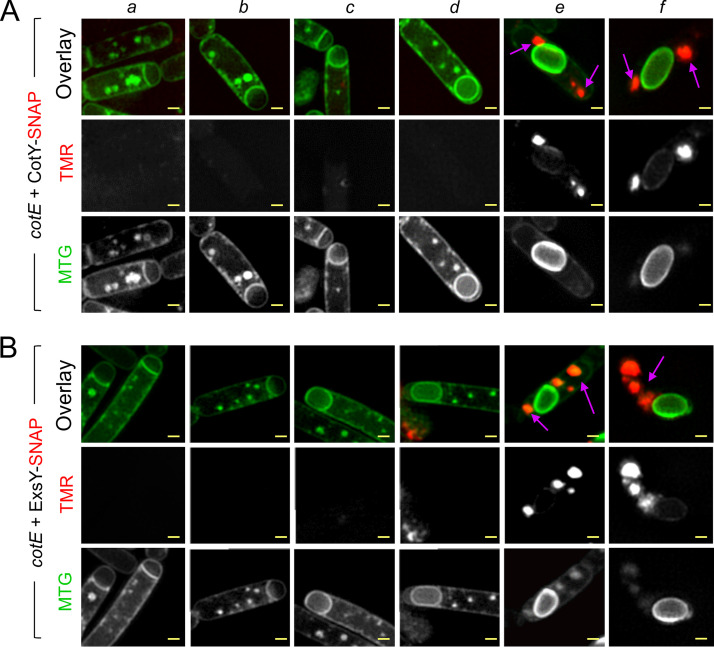
CotE is required for cap formation, allowing CotY and ExsY to localize as caps. Samples were collected from cultures of a *cotE* mutant producing CotY-SNAP (A) or ExsY-SNAP (B) during sporulation, and the cells were imaged by SR-SIM following staining with TMR-Star and MTG. Cells representative of the different localization patterns observed are shown (panels *a* to *f*). Purple arrows point to the signal from CotY-SNAP or ExsY-SNAP (panels *e* and *f*). In the *cotE* mutant, CotY-SNAP and ExsY-SNAP never formed the cap or the subsequent patterns identified in the WT (Fig. 1 and Fig. S1A) but formed large patches or aggregates in the mother cell cytoplasm. Bars, 0.5 μm.

We used SR-SIM to analyze the localization of CotY and ExsY in *cotE* sporangia and in mature spores. Neither cap formation nor encasement by CotY-SNAP or by ExsY-SNAP was observed in the absence of CotE (Fig. 2A and B, panels *a* to *f*). Remarkably, in the absence of CotE, CotY-SNAP accumulated as dots at the forespore poles, while ExsY-SNAP formed large aggregates in the mother cell, often close to the poles (Fig. 2A and B, respectively, purple arrows in panel *f*).

Since CotY and ExsY cap-shaped signals were never seen in *cotE* mutant sporangia, we reasoned that the cap could be absent in this strain, as reported for a B. anthracis *cotE* mutant (6). With TEM, we confirmed the absence of cap formation in B. cereus, and we found that in the absence of CotE, deposition of the coat follows a modified pathway (Fig. S4E to G and Text S1). Both fluorescence microscopy and TEM showed that CotE is required to form the MCP cap of the exosporium by allowing CotY and ExsY localization first as caps at the MCP forespore pole but then also for their localization as a second cap at the MCD forespore pole. This localization pattern and/or the presence of CotE appears to be important for guiding the subsequent encasement of the forespore by CotY and ExsY.

### ExsY is required for encasement by CotY but not for encasement by CotE.

**FIG 3:**
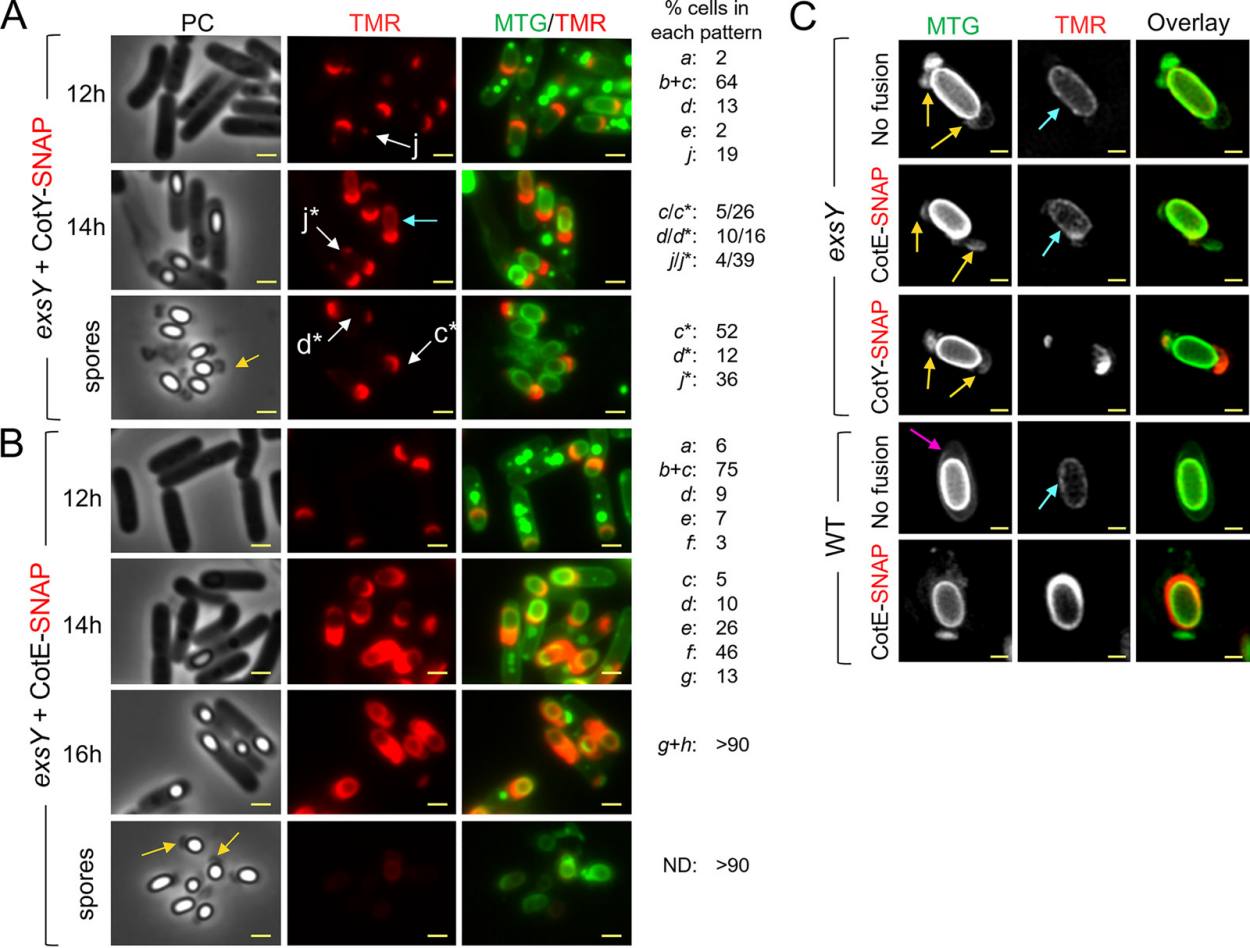
ExsY is required for encasement by CotY but not by CotE. (A and B) Samples were withdrawn at the indicated times from 37°C cultures of an *exsY* mutant producing CotY-SNAP (A) or CotE-SNAP (B). The cells were labeled with TMR-Star and MTG and were imaged by phase-contrast and fluorescence microscopy. Localization patterns *a* to *g* are identical to patterns *a* to *g* observed in WT cells in Fig. 1. Patterns c*, d*, and j* (white arrows) correspond to patterns *c*, *d*, and *j* observed in sporangia of phase-bright spores or in free spores. Numbers on the right show the percentage of cells with each of the indicated patterns relative to the total number of sporulating cells expressing the different SNAP fusions. ND; no signal detected. (C) Spores of the indicated strains were stained with MTG and TMR-STAR and were imaged by SR-SIM. The strains were ATCC 10876 (WT) and a derivative producing CotE-SNAP, as well as a congenic *exsY* mutant and derivatives producing CotE-SNAP or CotY-SNAP. Yellow arrows point to exosporia blocked at the two-cap stage. The pink arrow points to a complete exosporium. Cyan arrows point to nonspecific TMR signals. Bars, 1 μm in panels A and B and 0.5 μm in panel C.

CotY, ExsY, and CotE appeared to encase the forespore early in sporangia presenting only the cap region of the exosporium. Thus, we wondered whether the assembly of CotY and CotE is affected by the absence of ExsY, since only the cap region of the exosporium is assembled in B. anthracis *exsY* endospores (10). To test whether the localization of CotY and CotE requires ExsY, we used an *exsY* mutant previously obtained in ATCC 10876 (9). The amino acid sequences of B. cereus strains ATCC 10876 and ATCC 14579 are 100% identical for CotE and 92.9% identical for CotY. Moreover, we showed that the CotY-SNAP construction from the B. cereus ATCC 14579 CotY sequence complements the *cotY* mutation in ATCC 10876 (Fig. S5C). For this strain, however, for reasons we do not presently understand, when sporulation is induced at 20°C, engulfment is stopped in a fraction of the cells (not shown). We therefore induced sporulation at 37°C; in *exsY* mutant sporangia, CotY-SNAP localized as a cap at the onset of engulfment (Fig. 3A, pattern *b*) and until the end of engulfment (pattern *c*); together, these patterns represented 64% of the cells observed at hour 12. In engulfed sporangia at hour 12, CotY-SNAP formed a second, smaller cap at the MCD forespore pole (pattern *d*) in 13% of the cells and localized as a cap at the MCP pole and as a weak dot at the MCD pole (pattern *j*) in 19% of the cells. Importantly, the localization of CotY-SNAP did not change over time, suggesting a blockage of CotY assembly. Patterns *c*, *d*, and *j* were observed in phase-bright endospores and spores (Fig. 3A, patterns c*, d*, and j*). From hour 14, an inner signal was detected in phase-bright sporangia (Fig. 3A, cyan arrow), likely due to unspecific binding of the TMR to the forespore, as observed in a strain without a SNAP fusion (data not shown). In free spores observed at hour 34, CotY-SNAP never formed the fluorescent ring fully encasing WT spores. These results show, first, that as with B. anthracis, the MCP cap is assembled in a B. cereus *exsY* mutant. Second, they show that ExsY is required for asymmetric CotY encasement but not for CotY localization as two caps. Third, considering that the exosporium cap is made of CotY in *exsY* sporangia, these results suggest that CotY could form the basal layer of the cap independently of ExsY but relying on CotE.

10.1128/mSphere.00007-21.5 FIG S5 Localization of SNAP-CotE during sporulation of the *exsY* mutant. (A) Sporulating cells of an *exsY* mutant producing SNAP-CotE were collected at the indicated times and analyzed by fluorescence microscopy. Patterns *a* to *g* are similar to the ones described for the WT producing SNAP-CotE (Fig. 1 and Fig. S1B). Numbers on the right indicate the percentage of cells identified in each pattern (see Fig. 1 and the main text). ND, the fluorescence signal from SNAP-CotE is not detected. The yellow arrow points to the cap structure typically observed in *exsY* spores. Scale bar, 1 μm. Coat/exosporium extracts were prepared from spores of ATCC 10876 and the congenic *exsY* and *cotY* mutants (B) and derivative strains complemented through the ectopic production of ExsY-SNAP or CotY-SNAP (C), separated by SDS-PAGE, and subjected to immunoblotting with anti-CotE (bottom panels). The Coomassie-stained gels are shown as a loading control (top panels). One red asterisk indicates the CotE monomer; two red asterisks indicate a possible dimer; blue asterisks point to possibly cleaved forms; green asterisks point to nonspecific bands (see also Fig. S7). In panels B and C, the positions of molecular weight markers (MW, in kDa) are shown on the left. Download FIG S5, TIF file, 0.3 MB. Copyright © 2021 Lablaine et al. 2021 Lablaine et al. https://creativecommons.org/licenses/by/4.0/ This content is distributed under the terms of the Creative Commons Attribution 4.0 International license.

The sequences of CotE-SNAP and SNAP-CotE localization in *exsY* sporangia (Fig. 3B and Fig. S5A) were both similar to those in the WT (Fig. S1B and S2A). Interestingly, no signal from CotE-SNAP or SNAP-CotE was observed in the cap region of *exsY* spores after hour 34, despite a visible cap structure in phase-contrast images (Fig. 3B and Fig. S5A, yellow arrows). These observations suggest that CotE is not present or not detectable in *exsY* spores, although it assembles correctly during sporulation. A faint inner red TMR signal was observed, possibly corresponding to unspecific TMR binding to phase-bright forespores (Fig. 3B). To check this, we used SR-SIM to analyze *exsY* spores of strains producing CotY-SNAP or CotE-SNAP or *exsY* spores from a strain bearing no SNAP fusion. As suggested by conventional fluorescence microscopy (Fig. 3B), we detected only a faint internal TMR signal in *exsY* spores with CotE-SNAP (Fig. 3C, cyan arrow), which was also seen in WT spores and *exsY* spores without SNAP fusion. In spores of *exsY* producing either CotY-SNAP or CotE-SNAP and presenting a two-cap MTG signal (Fig. 3C, yellow arrows), we clearly observed fluorescence from CotY-SNAP-TMR (Fig. 3C), while no signal was observed for CotE-SNAP (Fig. 3C). This suggests that CotY was present in the cap of *exsY* mutant spores, while CotE was not (Fig. 3C). These results confirm the absence of a detectable signal from CotE-SNAP in spores lacking ExsY, even in the cap region. This observation suggests that CotE is required during sporulation only to guide cap assembly and that once the cap is formed by CotY, CotE is no longer required for the maintenance of cap integrity. These results also show that the arrested localization of CotY as a second cap in the MCD region of *exsY* sporangia gives rise to a second, smaller exosporium cap structure. We used immunoblotting to test for the presence or absence of CotE in *exsY* and *cotY* spores (Fig. S5B). In line with the microscopic analysis, CotE was not detected in *exsY* spores, but, more surprisingly, it was not detected in *cotY* spores, either (Fig. S5B).

Taken together, the localizations of CotE and CotY in the *exsY* mutant demonstrate that CotE encasement is independent of ExsY but also of CotY encasement, since ExsY is required for CotY encasement. Moreover, the absence of ExsY or CotY possibly affects the association of CotE with the released spores. Hence, since *cotY* spores possess a seemingly normal exosporium (7, 9), our results suggest that CotE is needed during sporulation, only to guide cap localization and encasement by ExsY and CotY, but seems dispensable later for maintaining the integrity of the structure once assembled. Finally, while CotE completely encased *exsY* forespores, CotY assembly was blocked at the two-cap stage. Thus, encasement by CotE is not sufficient to direct encasement by CotY, which additionally requires ExsY.

### CotE, CotY, and ExsY form a complex *in vivo* during sporulation and interact *in vitro*.

**FIG 4:**
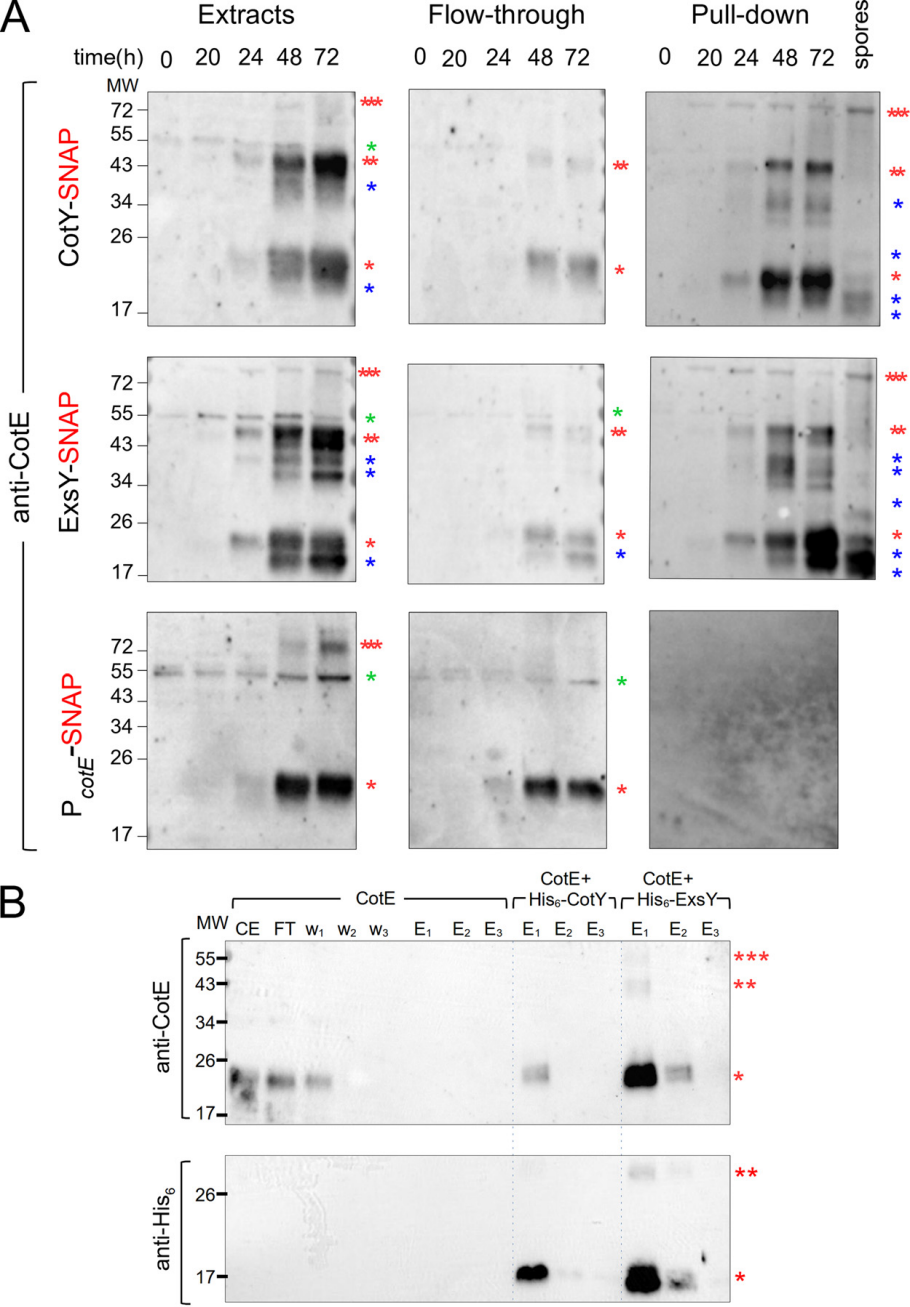
CotE, CotY, and ExsY form complexes *in vivo* and interact *in vitro*. (A) Samples were collected at the indicated times from sporulating cultures of B. cereus strains producing various SNAP fusions. Whole-cell extracts were prepared and subjected to pulldown assays with a SNAP capture resin. Whole-cell extracts, flowthrough, and bound proteins were resolved by SDS-PAGE and subjected to immunoblot analyses with anti-CotE antibodies. A lane corresponding to a pulldown assay performed on proteins extracted from purified CotY-SNAP and ExsY-SNAP spores was added. One red asterisk indicates a monomer of CotE; two red asterisks, a potential dimer of CotE; three red asterisks, multimers; blue asterisks, possible proteolytic products of CotE. A nonspecific signal in the extracts from vegetative cells (hour 0) is indicated by a green asterisk. (B) Heterologous coexpression pulldown assays. E. coli BL21(DE3) cells either producing CotE alone or coproducing CotE with His_6_-CotY or His_6_-ExsY were lysed and subjected to pulldown assays. Proteins were subjected to immunoblot analysis with anti-CotE (top) or anti-His_6_ (bottom) antibodies. While CotE produced alone was not eluted from the Ni^2+^ beads (6th to 8th lanes, top), His_6_-CotY pulled down CotE in E1 (9th lane, top) and was detected in E1 and E2 (9th and 10th lanes, bottom). CotE was pulled down with His_6_-ExsY in E1 and E2 (12th and 13th lanes, top) and was detected in E1 and E2 (12th and 13th lanes, bottom). CE, cell extract; FT, flowthrough; w_1_ to w_3_, washes; E_1_ to E_3_, elutions. Red asterisks indicate the different species of CotE (upper panels), His_6_-tagged CotY (9th to 11th lanes, bottom), or His_6_-tagged ExsY (12th to 14th lanes, bottom). The positions of molecular weight (MW) markers (in kDa) are indicated on the left.

Since CotE is required for the correct localization of CotY and ExsY and exhibits a similar sequence of localization during sporulation, we wondered whether CotE could form complexes with CotY and ExsY during spore formation. We used the ability of the SNAP tag to covalently bind SNAP capture agarose beads (33) to follow *in vivo* complex formation between CotE, CotY-SNAP, and ExsY-SNAP. Several of the proteins that form the exosporium are organized in complexes that resist solubilization (5, 8). Most of the exosporium proteins, however, assemble only late during sporulation (5). In addition, at an early time of sporulation, up to hour 24 in our experiments, only the MCP cap region of the exosporium is visible by TEM, while CotE, CotY, and ExsY already encase the forespore as a morphogenetic scaffold. Thus, in order to determine whether CotE is present in protein complexes involved in the early events of morphogenetic scaffold formation, pulldown assays with CotY-SNAP and/or ExsY-SNAP were conducted with extracts prepared from sporangia collected during engulfment (hour 20) and at the end of engulfment (hour 24). These studies were extended by performing pulldown assays with extracts from a mixture of phase-bright sporangia and spores (hours 48 and 72, respectively) and from purified spores collected at hour 72. In line with the microscopy observations, we observed accumulation of CotE (Fig. 4A, extracts), CotY-SNAP, and ExsY-SNAP (Fig. S6A, extracts) from hour 20 to hour 72 of sporulation, showing the better solubility of those proteins when sporulation takes place at 20°C. CotE was extracted as a species of about 20 kDa, corresponding to the expected size of the monomer; an abundant species of about 40 kDa, possibly corresponding to a dimer; and a less abundant multimeric form of about 75 kDa. We also detected species of intermediate apparent molecular weight, which may correspond to cleaved forms of CotE. SNAP fusion proteins were detected in the supernatant after the incubation of extracts collected from hour 48 with SNAP beads (Fig. S6A). All SNAP fusions were detected with anti-SNAP antibodies after the pulldown, and since the SNAP moiety binds covalently to SNAP capture beads, this suggests that the SNAP fusions could self-interact (Fig. S6A, pulldown). We found that CotY-SNAP and ExsY-SNAP pulled down the different forms of CotE extracted from sporulating cells and from purified spores (Fig. 4A, pulldown). Notably, the high-molecular-weight CotE species seems to be preferentially enriched in the pulldown compared to the extracts (Fig. 4A, three red asterisks). In contrast, a nonspecific species (Fig. 4A, green asterisk) detected from hour 0 (i.e., vegetative cells) was present in the extract and flowthrough fractions but absent in the pulldown. The SNAP tag alone, produced under the control of the *cotE* promoter (Fig. S6B, P*_cotE_*-SNAP fusion), did not retain CotE (Fig. 4A, pulldown). These results show that CotE formed complexes with CotY and with ExsY during sporulation and in mature spores of B. cereus. We also performed SNAP pulldown assays on extracts of sporulating *exsY* cells harboring CotY-SNAP, in which CotY assembly was blocked at the two-cap stage (Fig. 3A and Fig. S7B). The results indicate that CotE and CotY are present in complexes in the two caps and that the formation of these complexes is independent of the presence of ExsY (Fig. S7A and Text S1).

10.1128/mSphere.00007-21.6 FIG S6 CotY-SNAP, ExsY-SNAP, and SNAP (the latter produced under the control of the *cotE* promoter [P*_cotE_*-SNAP]) accumulate in cell extracts throughout B. cereus sporulation. (A) WT cells with the indicated SNAP fusions were harvested at the indicated times during sporulation. Extracts were prepared and used in pulldown assays and immunoblot analysis with anti-SNAP antibodies. An additional “spores” lane corresponds to pulldown done with extracts prepared from spores of the CotY-SNAP- and ExsY-SNAP-producing strains. One red asterisk indicates the indicated SNAP fusions; two or three red asterisks present potential multimeric forms of the SNAP-fusion proteins. The positions of molecular weight markers (MW, in kDa) are shown on the left. (B) Cells expressing P*_cotE_*-SNAP were collected at hour 24 of sporulation, labeled with TMR-Star and MTG, and imaged by phase contrast and fluorescence microscopy. The SNAP was detected dispersed throughout the mother cell cytoplasm. Scale bar, 1 μm. Download FIG S6, TIF file, 0.3 MB. Copyright © 2021 Lablaine et al. 2021 Lablaine et al. https://creativecommons.org/licenses/by/4.0/ This content is distributed under the terms of the Creative Commons Attribution 4.0 International license.

10.1128/mSphere.00007-21.7 FIG S7 CotE forms complexes with CotY in the cap of the *exsY* mutant. (A) Cells of an *exsY* mutant producing CotY-SNAP were harvested at hours 0, 24, 28, 48, and 72 of sporulation, and whole-cell extracts were subjected to pulldown and immunoblot analysis with anti-CotE and anti-SNAP antibodies. One red asterisk indicates CotE or a CotY-SNAP monomer; two red asterisks indicate potential multimers; blue asterisks point to possible products of proteolysis; green asterisks point to nonspecific bands reacting with the anti-CotE antibody on vegetative cell extracts. The positions of molecular weight markers (MW, in kDa) are shown on the left. (B) CotY-SNAP localization in the *exsY* mutant at 28, 48, and 72 h of sporulation at 20°C. The numbers on the right of the panels indicate the percentage of cells identified in each pattern (see Fig. 1 and the main text). The panels show the CotY-SNAP signal blocked at the cap stage. Scale bar, 1 μm. Download FIG S7, TIF file, 0.3 MB. Copyright © 2021 Lablaine et al. 2021 Lablaine et al. https://creativecommons.org/licenses/by/4.0/ This content is distributed under the terms of the Creative Commons Attribution 4.0 International license.

To determine if CotE interacts directly with CotY and/or ExsY, the proteins were coproduced in Escherichia coli and tested for pairwise interactions (34). Coexpression using the pETDuet system allows interactions to occur in the cell and the purification of protein complexes (34). Notably, organized supramolecular structures formed by B. subtilis coat proteins were purified with this approach (21). To determine possible interactions, we coexpressed CotE with His_6_-CotY or His_6_-ExsY in E. coli. We observed that CotE was extracted as monomers when produced alone (Fig. 4B, lane CE), while multimeric forms were present when CotE was coproduced with His_6_-CotY or His_6_-ExsY (data not shown). We observed that His_6_-CotY, His_6_-ExsY, and CotE accumulated in E. coli and were correctly detected by the appropriate antibodies. CotE, when produced alone, was detected in the extracts (Fig. 4B, lane CE, top), in the flowthrough (Fig. 4B, lane FT, top), and in the first wash (Fig. 4B, lane w_1_, top) and was not retained by the Ni^2+^ beads after washes (Fig. 4B, lanes E_1_ to E_3_, top). When coproduced with His_6_-ExsY, CotE was eluted in large amounts mainly in E_1_ (including multimeric forms [two or three red asterisks]) and partly in E_2_ (Fig. 4B, lanes E_1_ and E_2_, top). When CotE was coproduced with His_6_-CotY, only a small fraction of CotE was eluted in E_1_ (Fig. 4B, lane E_1_, top, one red asterisk). These results show that CotE clearly interacts with ExsY and interacts slightly with CotY.

## DISCUSSION

**FIG 5:**
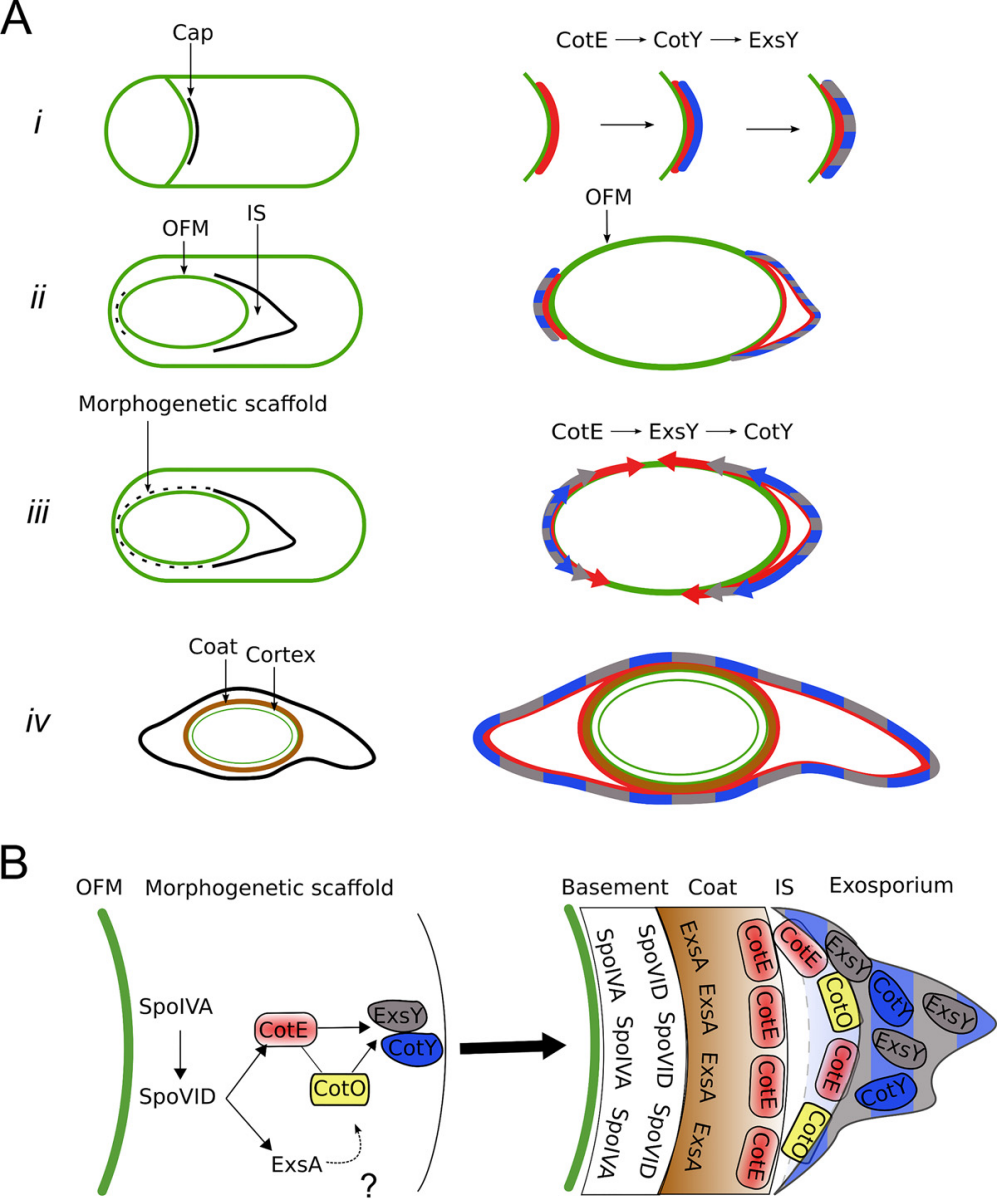
Successive localization, interactions, and interdependence among CotE, CotY, and ExsY during exosporium formation. (A) (Left) Assembly of the indicated spore structures as observed by TEM. Dotted lines show the new structures found in the present study. (Right) Diagrams showing the temporal sequence of localization of CotE, CotY, and ExsY inferred from our results. (*i*) CotE (red) forms a cap in the septal region at the onset of engulfment and recruits CotY (blue), which, in turn, recruits ExsY (gray). Once positioned, CotY and ExsY form the basal layer of the cap (mixed gray and blue). The cap remains unchanged until the completion of engulfment. (*ii*) After the completion of engulfment, the MCP cap is separated from the OFM by the formation of the interspace (IS), and CotE directs the localization of CotY, and therefore of ExsY, as a second cap on the MCD pole (dotted line). (*iii*) CotE progressively encases the spore starting from one longitudinal side of the forespore (red arrows), guiding the simultaneous encasement of ExsY (gray arrows), which, in turn, is required for the encasement of CotY (blue arrows). After the completion of encasement by CotE, CotY and ExsY cover the noncap region of the forespore as an immature morphogenetic scaffold (dotted line). (*iv*) After coat formation and insertion of the late-synthesized proteins, CotE, CotY, and ExsY are found in all the regions of the mature exosporium. (B) Formation of the morphogenetic scaffold and its maturation in B. cereus. Based on their roles in B. subtilis, SpoIVA and SpoVID homologs are good candidates for directing the recruitment and encasement, respectively, of CotE and CotE-controlled proteins. CotO, possibly through its interaction with CotE, may participate in the recruitment of CotY and/or ExsY. ExsA, a SafA homologue that controls B. cereus coat protein deposition, is also present in the morphogenetic scaffold. After sporulation completion, the proteins of the morphogenetic scaffold are positioned in the different layers of the mature spore.

Here, we examined the assembly of the exosporium in B. cereus, focusing on CotY and ExsY and on their relationship with CotE. We show that CotY, ExsY, and CotE share a common pattern of localization and are detected as complexes throughout sporulation and that CotE can interact directly with CotY and ExsY. CotY, ExsY, and CotE colocalize at the MCP forespore pole during engulfment, forming the MCP cap structure (Fig. 5A*i*). After the completion of engulfment, the three proteins colocalize as a second cap at the MCD forespore pole (Fig. 5A*ii*). CotE is required for the localization of both ExsY and CotY as a cap at both poles; however, their MCD cap localization does not give rise to a structure detectable by TEM (Fig. 5A*ii*, dotted line). In the absence of CotE, CotY-SNAP forms dots at the forespore poles and ExsY-SNAP becomes dispersed throughout the mother cell cytoplasm. Finally, from the two caps, the three proteins encase the forespore asymmetrically, covering one longitudinal side after the other (Fig. 5A*iii* and *iv*). Thus, the assembly of the B. cereus exosporium does not seem to be unidirectional, as had been deduced from B. anthracis studies (5, 12, 35).

Certain aspects of this pathway deserve special attention. With SR-SIM, we were able to distinguish the cap part of the exosporium at the time of its separation from the OFM by the formation of the interspace, just after engulfment completion. We show that at this stage in morphogenesis, CotY, ExsY, and CotE migrate from the OFM to form the visible basal layer of the cap region of the exosporium and to start the encasement of the forespore (Fig. 5A*ii* and *iii*). How the separation of CotY, ExsY, and CotE to form the cap is brought about is unclear. With a CotE-SNAP fusion, we noticed that the cap was closer to the OFM, suggesting that the SNAP tag interferes with a function of the C-terminal region of CotE in the separation of the cap from the OFM and, more generally, that CotE may be implicated in interspace formation. However, this question, often raised (5, 22), has to be further investigated.

The localization of CotE, CotY, and ExsY at the MCP forespore pole gives rise to a visible separated MCP cap structure observed by SR-SIM and TEM. In contrast, we show that these proteins are already localized as a second cap at the MCD forespore pole, while this exosporium region is detectable by TEM only after coat assembly begins (Fig. 5A*ii*, dotted line). We detected a second cap structure by SR-SIM in *exsY* sporangia only, in which CotY-SNAP encasement is blocked. This second cap, not observed by TEM in B. anthracis *exsY* sporangia (10), is observed in *exsY* spores (without SNAP fusion) and in *exsY* spores with CotY-SNAP, whose fluorescent signal colocalizes with that from ExsY-SNAP inside the exosporium MCD cap structure. Notably, the CotY-SNAP signal at the MCD cap is often weaker than the signal from the MCP cap (pattern *j*) and is detected only in half of the free spores, while most of the sporangia present an MCD signal. This suggests that the MCD cap is less connected than the MCP cap to *exsY* spores and possibly dissociates when the spore is released. This presumably explains why the MCD cap was not reported by TEM, a more-disruptive technique, in previous studies (10).

The last events in the assembly of CotY, ExsY, and CotE around the forespore follow a particular dynamic, in that a three-quarters-of-a-circle localization pattern (pattern *e*) is detected. While CotY-SNAP localized as two caps (pattern *d*) in *exsY* sporangia, pattern *e* was not observed, showing that the localization as a three-quarter circle occurred after the localization as two caps and that this localization of CotY relies on ExsY. In addition, since the three-quarter-circle localization of CotE is independent of ExsY, it seems that CotE imposes this localization on ExsY and thus on CotY (Fig. 5A*iii*). Recently, Boone et al. reported that the assembly of the exosporium in a *cotY* mutant, and therefore the assembly of ExsY, initiates at random locations (7). Based on our results, we propose that the encasement of the forespore by ExsY in a *cotY* mutant is allowed by a CotE-ExsY interaction, since asymmetric CotE encasement appears to be independent of CotY encasement. Therefore, it appears that CotE-ExsY interaction can bypass the possible requirement of CotY for ExsY recruitment.

Importantly, encasement by CotY, ExsY, and CotE was completed before the appearance of the noncap part of the exosporium or coat formation, as observed by TEM. We propose that these proteins form a morphogenetic scaffold at the surface of the noncap part of the forespore. Previous studies suggested that a region named the spore-free sacculus, or “sac,” is implicated in exosporium assembly, allowing the attachment of the cap in *exsY* sporangia as well as the assembly of ExsY (8, 10, 22). The morphogenetic scaffold that we describe here seems to correspond to this “sac” and relies largely on the interactions of CotE with CotY and ExsY during exosporium formation.

The assembly pathway of CotY, ExsY, and CotE is reminiscent of that of class II coat proteins in B. subtilis, which localize as an MCP cap and an MCD cap following engulfment completion when encasement starts. There are, however, differences to be highlighted. B. subtilis CotZ is a class III protein, i.e., it begins encasement only when the forespore turns phase-dark. However, in B. subtilis, but not in B. cereus, the transcription of *cotY* and *cotZ* is under the control of GerE, which could explain the different behavior, since encasement also depends on waves of gene expression (20, 36). Finally, in the absence of CotZ, B. subtilis CotY fails to form a second cap (37).

Nevertheless, the assembly of the spore surface layers may follow common principles across species. The B. subtilis coat initially assembles as a scaffold composed of proteins present in the four layers of the mature coat, including SpoIVA, SpoVID, SafA, CotE, and CotZ. SpoIVA recruits proteins to the spore surface; SpoVID controls encasement; SafA controls the assembly of the inner coat; CotE directs the assembly of the outer coat; and together, CotE and CotZ govern the assembly of the crust (18–20). SpoIVA and SpoVID are conserved in the B. cereus group, where a *spoIVA* mutation leads to important defects of exosporium and coat assembly, as in B. subtilis (6, 22). It is likely that SpoIVA and SpoVID have similar roles in the B. cereus group (Fig. 5B). Recently, it was shown that the GerP coat proteins did not localize properly in a B. cereus *exsA* mutant but localized normally in a *cotE* mutant, suggesting separate control of coat and exosporium assembly (38). ExsA, a paralogue of B. subtilis SafA, is transcribed just after the initiation of sporulation, and while an *exsA* mutation impairs exosporium assembly, this is likely an indirect effect due to the misassembly of the internal coat (39). Thus, ExsA may also be present in the morphogenetic scaffold of B. cereus that we propose here (Fig. 5B) and may have a role equivalent to that of SafA in B. subtilis (40). Also recently, in B. anthracis, CotO was shown to share similar characteristics with CotY and ExsY. CotO is an orthologue of a class II morphogenetic protein implicated in the encasement of the spore by crust proteins in B. subtilis (20, 37). B. anthracis CotO interacts with CotE, is dependent on CotE for its localization, and shows potential class II localization kinetics (7). Thus, it is tempting to propose that CotO is part of the morphogenetic scaffold of B. cereus together with CotE, CotY, and ExsY (Fig. 5B). Interestingly, CotO is required for the assembly of the exosporium, and *cotO* mutation leads to a phenotype similar to that of *cotE* mutation (7), suggesting that CotE and CotO work in concert, possibly by stabilizing the complexes formed by CotE, CotY, and ExsY that we describe here.

The formation of the coat scaffold of B. subtilis and the dynamics of the coat/crust proteins are driven by self-polymerization and by direct interactions (21, 24). Similarly, it has been shown that CotY and ExsY could self-polymerize in B. cereus (8). We found that ExsY polymerization seems not to be dependent on CotE and is not required for CotE assembly around the spore but appears necessary for the assembly of CotY. The cross-linking, via formation of disulfide bonds, between ExsY monomers and with CotY, could be a driver of exosporium formation. The resulting ExsY/CotY expansion around the developing spore is guided by a sublayer of CotE, which, in turn, may rely on a SpoIVA/SpoVID platform. Thus, CotE and the CotE-controlled proteins, CotO, CotY and ExsY, are recruited in the B. cereus morphogenetic scaffold (Fig. 5B).

The incorporation of a second group of late-synthesized proteins, such as CotB, ExsB, ExsK, BxpB, BetA, or BclA, could allow the maturation of the coat and exosporium structures (3, 15, 16, 36, 41–44). Most of these proteins are unique to the B. cereus group and are likely responsible for the distinctive properties of the spore surface layers (22). In any case, regardless of the important structural differences observed between the B. subtilis and B. cereus proteinaceous layers, a scaffold made of morphogenetic proteins fulfills the same role in both by guiding the localization of other components through direct protein-protein interactions.

## MATERIALS AND METHODS

### Bacterial strains and growth conditions.

The bacterial strains and plasmids used in the present study are listed in Table S1. LB broth with orbital shaking (200 rpm) or LB agar plates were used for routine growth of B. cereus and E. coli at 37°C. When needed, liquid cultures or plates were supplemented with the following antibiotics at the indicated concentrations: ampicillin (Amp) at 100 μg·ml^–1^ for E. coli cultures, spectinomycin (Spc) at 275 μg·ml^–1^, and erythromycin (Erm) at 5 μg·ml^–1^ for B. cereus cultures.

10.1128/mSphere.00007-21.8 TABLE S1 Strains, plasmids, and primers used in this study. Download Table S1, DOCX file, 0.04 MB. Copyright © 2021 Lablaine et al. 2021 Lablaine et al. https://creativecommons.org/licenses/by/4.0/ This content is distributed under the terms of the Creative Commons Attribution 4.0 International license.

### Other general methods.

The construction of SNAP fusions and plasmids for the coproduction of proteins in E. coli, as well as the methods used for protein coproduction, pulldown assays, and immunoblot analysis, are described in Text S2 in the supplemental material.

10.1128/mSphere.00007-21.10 TEXT S2 Supplemental material and methods. Download Text S2, DOCX file, 0.03 MB. Copyright © 2021 Lablaine et al. 2021 Lablaine et al. https://creativecommons.org/licenses/by/4.0/ This content is distributed under the terms of the Creative Commons Attribution 4.0 International license.

### Sporulation kinetics.

Sporulation was induced in liquid SMB medium at either 20°C or 37°C with orbital shaking at 180 rpm, as described previously (23). Cells were harvested at the desired time by centrifugation for 10 min at 10,000 × *g* before microscopy or immunoblot analysis. When necessary, spores were collected after 72 h of incubation and were purified by successive centrifugations and washes with cold water as described elsewhere (23). For immunoblot analysis, B. cereus spores were also produced at 20°C on modified fortified nutrient agar (mFNA) (23).

### SNAP labeling and fluorescence microscopy.

Samples (5 to 10 ml) were withdrawn from cultures in SMB at selected times during sporulation. Cells were collected by centrifugation (10,000 × *g* for 10 min), suspended in 200 μl of phosphate-buffered saline (PBS), and labeled with TMR-Star (New England Biolabs) for 30 min at 37°C in the dark at a final concentration of 250 nM. This TMR-probed suspension was centrifuged (12,000 × *g*, 3 min); then the cell sediment was washed with 1 ml of PBS, suspended again in 1 ml of PBS, and labeled with MitoTracker Green (MTG; Thermo Fisher) for 1 min at room temperature at a final concentration of 1 μg/ml. The cells were then washed three times in PBS and suspended in 50 μl to 200 μl PBS, depending on the concentration of sporulating cells. For phase-contrast and fluorescence microscopy, a 3-μl volume of the labeled cell suspension was applied to 1.7% agarose-coated glass slides and observed with an epifluorescence microscope (BX-61; Olympus) equipped with an Orca Flash 4.0 LT camera (Hamamatsu). Images were acquired using CellSens Olympus software, and micrographs were processed using ImageJ software. For quantification of the subcellular localization of SNAP fusions, a minimum of 150 cells were counted, and the different patterns identified were randomly examined and scored. Super-resolution structured illumination microscopy (SR-SIM) images were acquired using an Elyra PS.1 microscope (Zeiss) equipped with a Plan-Apochromat 63×/1.4 oil DIC M27 objective and a Pco. edge 5.5 camera, using 488-nm (100-mW) or 561-nm (100-mW) laser lines, at 5 to 20% of total potency (27). The grid periods used were 23 mm, 28 mm, or 34 mm for acquisitions with the 488-nm or 561-nm lasers. For each SR-SIM acquisition, the corresponding grating was shifted and rotated five times, giving a total of 25 acquired frames. Final SR-SIM images were reconstructed using ZEN software (black edition, 2012, version 8,1,0,484; Zeiss), using synthetic, channel-specific optical transfer functions (OTFs). At least 100 sporangia/30 spores produced at 20°C were examined at each sampling time. The distance between the cap and the forespore membranes was evaluated on at least 34 sporangia. All the kinetics were replicated at least twice using conventional fluorescence microscopy before being imaged once by SR-SIM.

### Transmission electron microscopy.

Sporulating cells were centrifuged at 3,500 × *g* for 5 min and were fixed for 1 h at room temperature and overnight at 4°C with 5% (vol/vol) glutaraldehyde in 0.1 M sodium cacodylate buffer (pH 7.2) containing 1 mg·ml^–1^ ruthenium red. Cells suspended in 0.2 M sodium cacodylate were subjected to three successive centrifugations (5 min at 3,500 × *g*) and supernatant elimination and postfixed for 1 h at room temperature with 2% osmium tetroxide. Cells were observed by TEM (Hitachi HT7800). A minimum of 30 sporangia were examined at the different sampling times. The distance between the cap and the forespore membranes was scored on at least 15 sporangia (except for CotY-SNAP [*n* = 11]).
