# Supplementary figures and images for: The Morphogenetic Protein CotE Positions Exosporium Proteins CotY and ExsY during Sporulation of Bacillus cereus
Source: mSphere. 2021 Apr 21;6(2):e00007-21. doi: 10.1128/mSphere.00007-21 (PMC8546674; doi:10.1128/mSphere.00007-21)

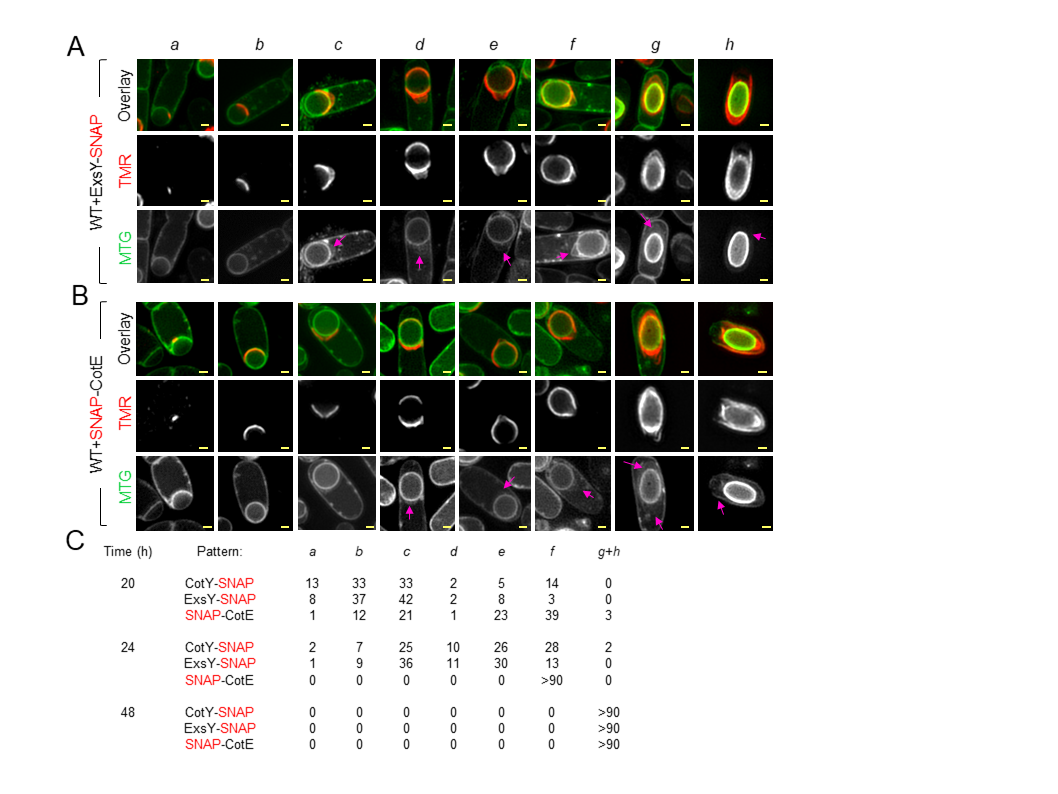

Supplement: FIG S1 [file msphere.00007-21-sf001.tif]

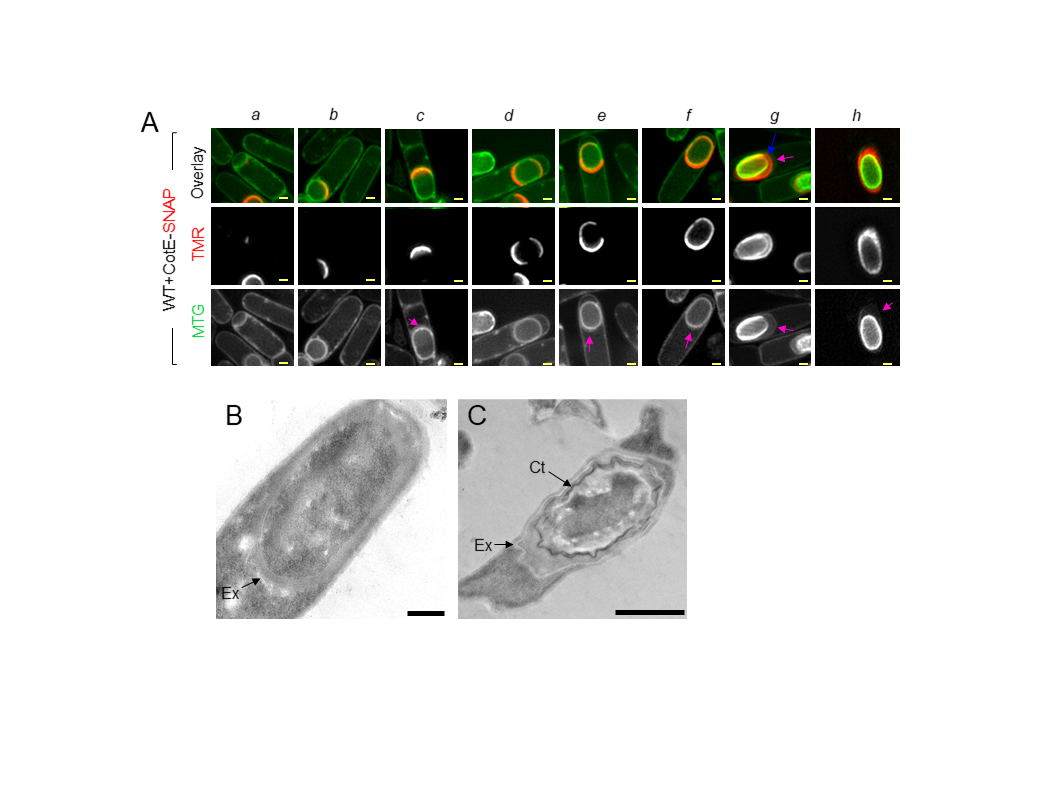

Supplement: FIG S2 [file msphere.00007-21-sf002.tif]

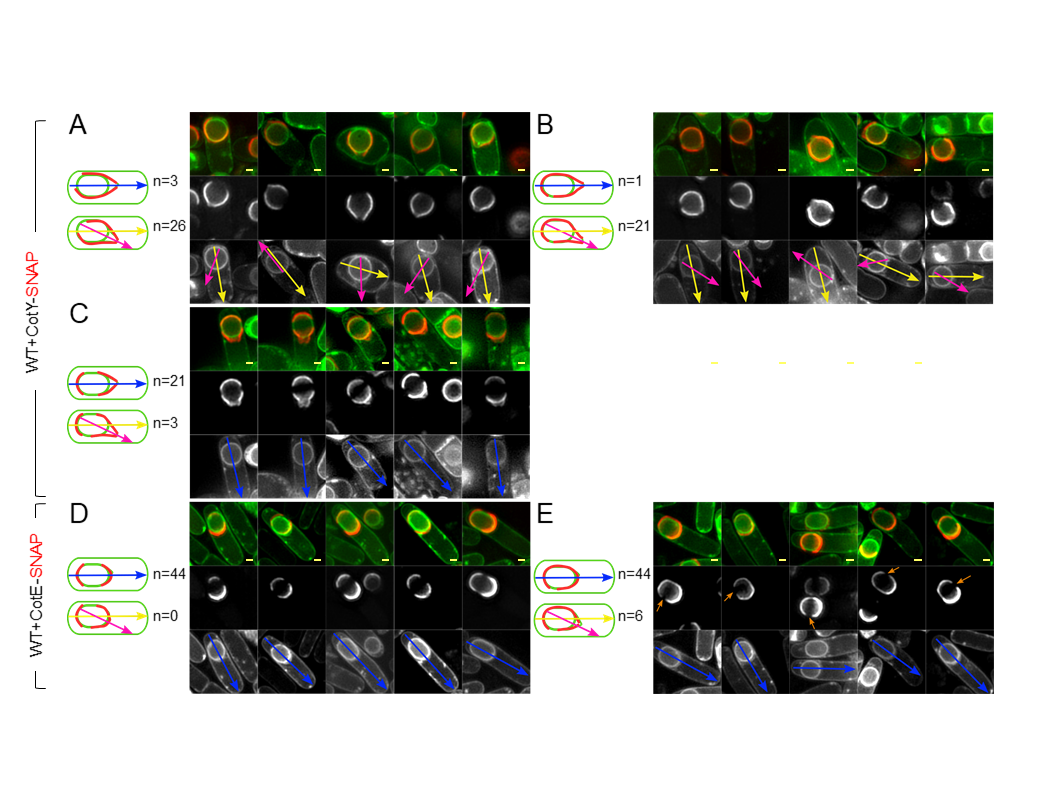

Supplement: FIG S3 [file msphere.00007-21-sf003.tif]

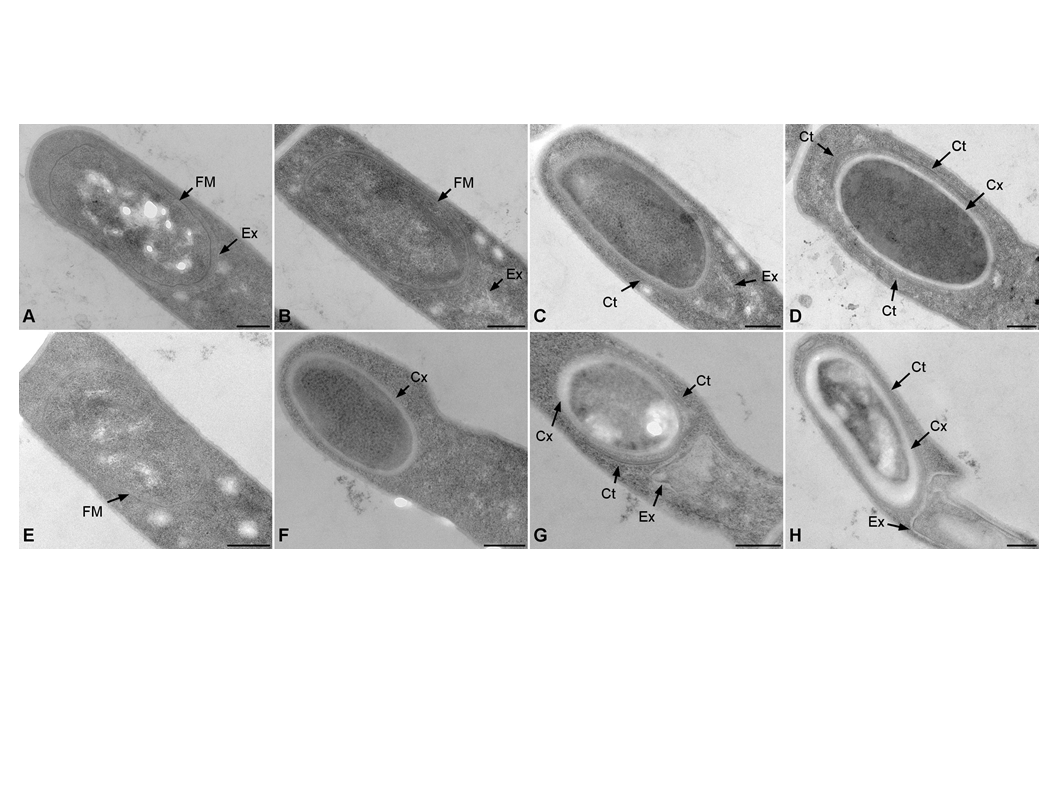

Supplement: FIG S4 [file msphere.00007-21-sf004.tif]

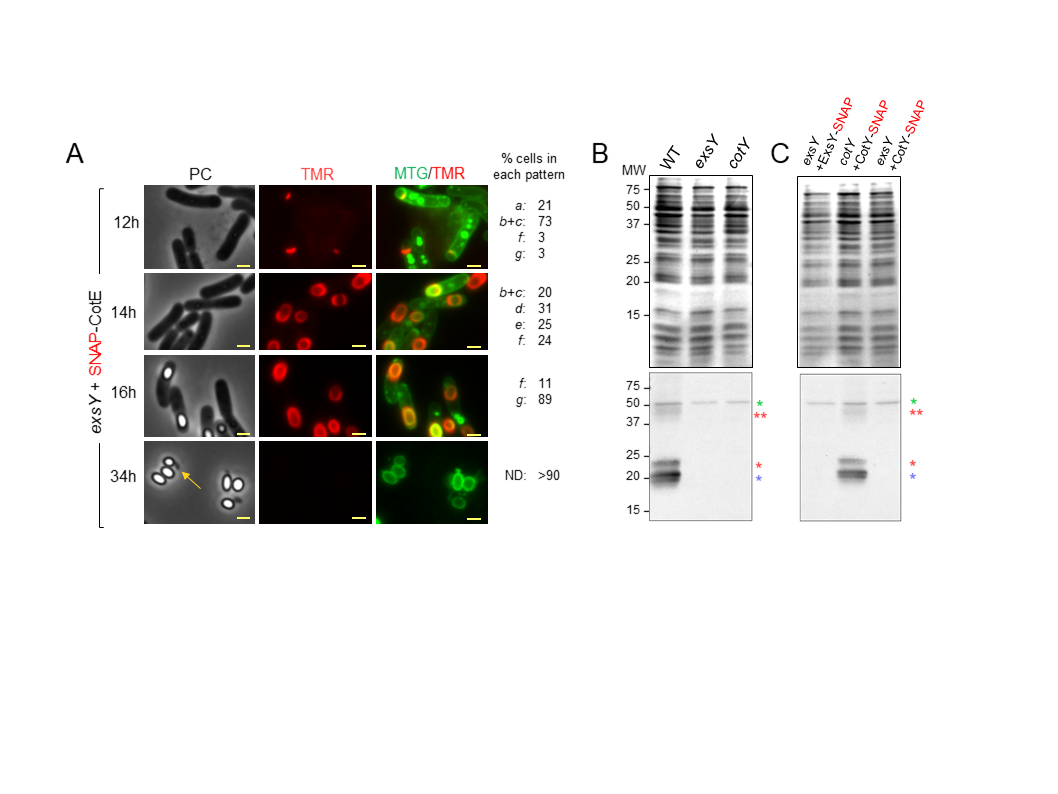

Supplement: FIG S5 [file msphere.00007-21-sf005.tif]

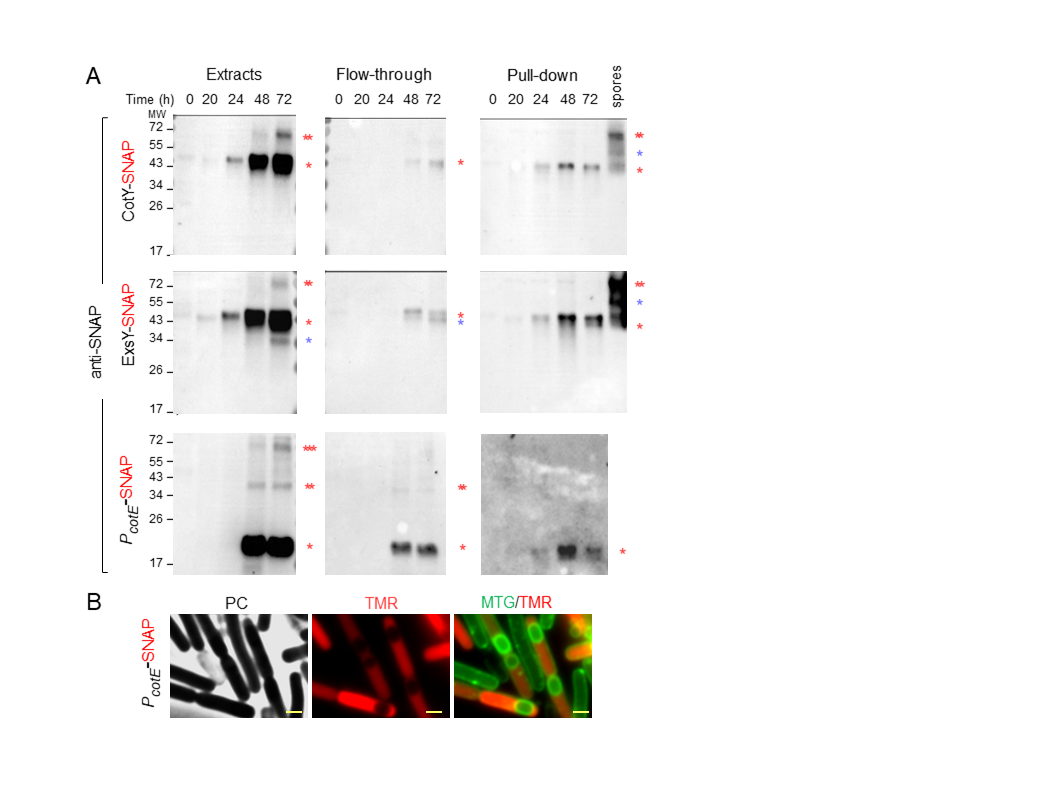

Supplement: FIG S6 [file msphere.00007-21-sf006.tif]

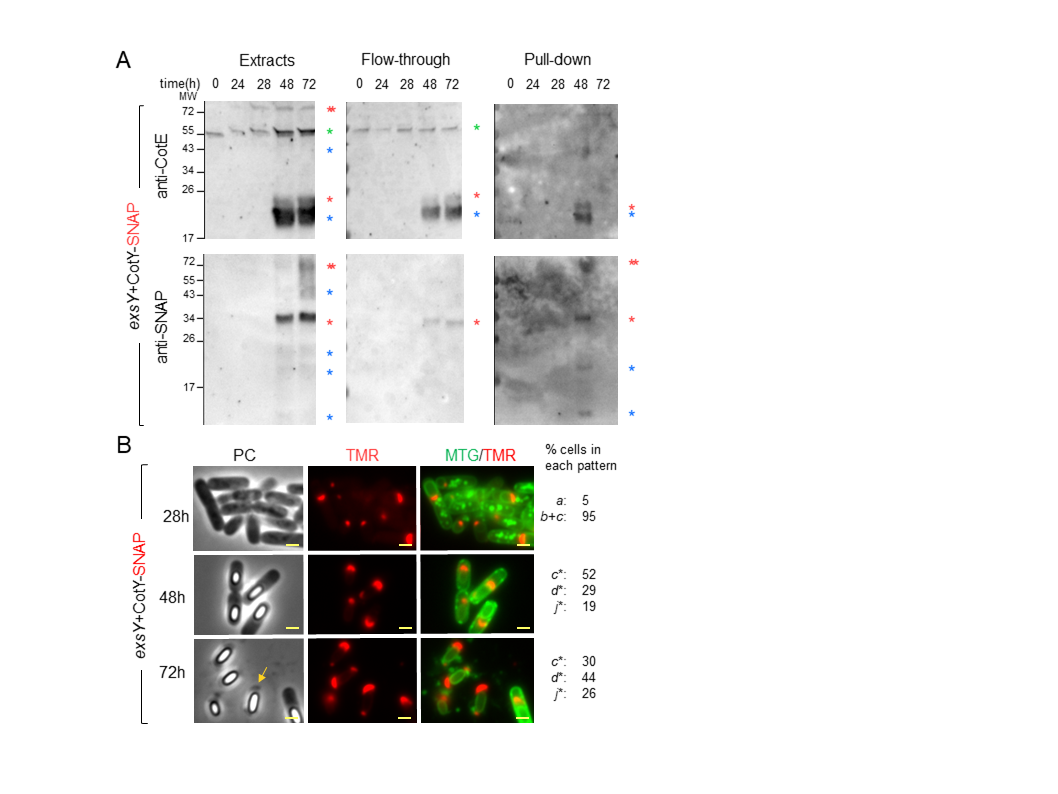

Supplement: FIG S7 [file msphere.00007-21-sf007.tif]
