# Supplementary material for: The Morphogenetic Protein CotE Positions Exosporium Proteins CotY and ExsY during Sporulation of Bacillus cereus
Source: mSphere. 2021 Apr 21;6(2):e00007-21. doi: 10.1128/mSphere.00007-21 (PMC8546674; doi:10.1128/mSphere.00007-21)
Supplement: TEXT S1 [file msphere.00007-21-s0001.docx]

**SUPPLEMENTAL TEXT**

**The morphogenetic protein CotE positions exosporium proteins CotY and ExsY during sporulation of *Bacillus cereus***

Armand Lablaine^a^, Monica Serrano^b^, Christelle Bressuire-Isoarda, Stephanie Chamot^a^, Isabelle Bornard^c^, Frederic Carlin^a^, Adriano O. Henriques ^b*^ and Veronique Broussolle ^a*^

^a^ INRAE, Avignon Université, UMR SQPOV, F-84000 Avignon, France

^b^ Instituto de Tecnologia Quimica e Biologica, Universidade Nova de Lisboa, 2780-157 Oeiras, Portugal

^c^ INRAE, Pathologie végétale, F-84143 Montfavet, France

* address correspondence to Veronique Broussolle, [veronique.broussolle@inrae.fr](mailto:veronique.broussolle@inrae.fr) and Adriano O. Henriques, aoh@itqb.unl.pt

Running title: Assembly of *Bacillus cereus* exosporium

Keywords: Spore, morphogenetic proteins, exosporium, SR-SIM

**SUPPLEMENTAL RESULTS AND DISCUSSION**

**A role for CotE in separation of the cap from the forespore.** Previous proteomic studies reported the presence of a cleavage product of CotE corresponding to the N-terminal domain of the protein in the exosporium basal layer of *B. cereus* (1, 2). Here we noticed that the fluorescence signal from CotE-SNAP (*i.e.*, with the SNAP-tag fused to the C-terminal end of CotE) slightly differs from the signal obtained with SNAP-CotE (with the SNAP-tag fused to the N-terminal end of CotE) in cells having completed engulfment (Fig. S2A): notably, we did not observe migration of CotE-SNAP from the OFM into the MCP exosporium cap, as seen for CotY-, ExsY-SNAP and SNAP-CotE (Fig. 1 and Fig. S1). We consider two possibilities: i) CotE domains may be partitioned between the exosporium and the coat, or ii) the exosporium cap may not correctly separate from the OFM after engulfment completion. In trying to answer this question, we analyzed 46 SR-SIM images of post-engulfed WT cells producing CotE-SNAP: we estimated that the average distance between the external limit of the MTG cap signal and the signal of the forespore membranes was 180±38 nm (mean ± sd, n=46) (Fig. S2A, panels *c* to *f*, pink arrows) and therefore was less than that measured in WT post-engulfed cells without SNAP fusion 238±70 nm, (n=34; not shown) or in WT cells producing CotY-SNAP (242±79 nm, n=72; Fig. 1). However, while the signals from CotY-SNAP, ExsY-SNAP and SNAP-CotE clearly appeared in the exosporium layer labeled with MTG in sporangia imaged at hour 48 (Fig. 1, S1A and B, panels *g* and *h*), CotE-SNAP seemed to be present in a more internal layer, distinct from the exosporium (Fig. S2A, panel *g*, blue arrow). These differences suggest that the CotE-SNAP fusion interferes with the separation of the cap from the forespore membranes. Additionally, on TEM images of CotE-SNAP sporangia that had completed engulfment (Fig. S2B), we never observed the separated cap structure seen in WT sporangia (Fig. S4C). Based on measurements made on 22 TEM images of post-engulfed CotE-SNAP sporangia, we estimate that the average distance between the external limit of the cap from the forespore membranes was only of 83±38 nm (Fig. S2B), shorter than in WT cells (147± 51nm, n=26; Fig. S4C) or in CotY-SNAP sporangia (161±27nm, n=11; not shown). Together those results show that the presence of the CotE-SNAP fusion in the WT background interferes with the separation of the exosporium cap after engulfment completion. However, as the wild-type CotE copy is also present and may carry out normal interactions, this interference seems surprising. Morphogenetic coat proteins are known to interact with multiple partners (3-7), notably *B. anthracis* CotE interacts with CotO and the complex formed by those proteins is proposed to be involved in the separation of the exosporium from the coat (8). Thus, if the addition of the SNAP-tag at the C-terminal domain of CotE impairs CotE interactions with partners, the interaction of CotE with CotE-SNAP may prevent CotE to interact with the other partners by antagonizing the site of interactions. Supporting this hypothesis, we observed that CotE-SNAP pulls down CotE during sporulation (data not shown). However, a redundant mechanism seems to allow interspace formation in the MCP region, as we observed a normal exosporium in phase-bright sporangia expressing CotE-SNAP collected at hour 48 (Fig. S2C). In the non-cap region of the exosporium, the interspace is formed later, at the time of coat deposition (9), thus the MCP cap may be separated at this later time in CotE-SNAP sporangia. Altogether, those results show that the production of CotE-SNAP in a WT background interferes transiently with the separation of the MCP cap from the OFM, in a way we presently do not fully understand.

**The encasement by the proteins of the morphogenetic scaffold is bidirectional and asymmetric.** After engulfment completion and before the forespore turns phase bright and coat is detected by TEM, CotE-, CotY- and ExsY-SNAP show heterogeneous localization patterns, meaning that encasement by those proteins is a rapidly evolving process (Fig. 1 and S1, patterns *c* to *f*). Importantly, the two caps localization (pattern *d*) may indicate that the assembly process of CotE-, CotY- and ExsY-SNAP can be bidirectional, as observed during encasement by CotE, CotY and CotZ *B. subtilis* orthologues (10-11). This is quite unexpected, as in *B. anthracis*, exosporium assembly is seen as unidirectional, starting from the MCP cap and progressing towards the MCD forespore pole. Furthermore, TEM analysis of *B. cereus* sporangia supports the same unidirectional exosporium assembly (9, 12-14).

To better understand how CotE, CotY and ExsY encase the forespore after engulfment completion, we analyzed specifically SR-SIM images of CotY-SNAP sporangia with an identifiable exosporium MCP cap and advanced patterns of CotY-SNAP encasement, *i. e* patterns *d* or *e* (Fig S3A-C, n=75). Surprisingly, we noticed different orientations of the exosporium MCP cap structure (pink arrows) regarding the axis of the mother cell (MC, yellow arrows). The exosporium MCP cap was aligned with the MC longitudinal axis (blue arrows) in 25 out of 75 sporangia observed, and was off-center in a majority of sporangia (50 out of 75). Most of the sporangia with an off-center MCP cap (47 out of 50 cells, panels A and B) showed a ¾ of a circle localization of CotY-SNAP, with a MCD forespore pole encased (Fig. S3B, 21 out of 50 sporangia) or not encased by CotY-SNAP (Fig S3A, 26 out of 50). In contrast, among the 25 sporangia with a MCP cap structure aligned with the axis of the MC (Fig. S3A-C, blue arrows), 21 showed a typical two caps-localization pattern (Fig. S3C), while only 4 presented a ¾ of a circle localization pattern (not shown and Fig. S3A-B). The same observations were obtained for ExsY-SNAP and SNAP-CotE sporangia (data not shown), suggesting that the MCP cap formed by CotE, CotY and ExsY can be mobile around the forespore.

The apparent mobility of the proteins forming the exosporium cap around the forespore makes it difficult to draw any conclusions regarding the final step of encasement by those proteins. However,
we speculate that the offset of the MCP cap observed in cells expressing CotY-, ExsY-SNAP and SNAP-CotE should require the separation of the cap from the OFM by the interspace formation. Thus, as we reported that the expression of CotE-SNAP in WT cells precisely affects this mechanism, which normally occurs after engulfment completion, we deeply investigated the encasement by CotE-SNAP. We identified 94 engulfed sporangia showing an aggregated MTG signal close to the OFM, corresponding to the connected MCP cap, as reported in Fig. S2 (pink arrow) and showing a two caps localization (Fig. S3D) or a ¾ of a circle localization of CotE-SNAP (Fig. S3E). Only 6 of these cells presented an off-center MCP cap structure which, additionally, appeared normally separated from the OFM (data not shown). Importantly, 88 out of 94 sporangia show a MCP exosporium cap aligned with the MC axis (panels E-F, blue arrows). This result confirms that the changes of exosporium MCP cap alignment observed in SNAP-CotE, CotY- or ExsY-SNAP cells (Fig. S3A-B and not shown) required the separation of the cap from the OFM. Moreover, half of the counted sporangia with an exosporium cap in the MC axis presented a typical two caps-localization of CotE-SNAP (Fig. S3D) and the other half showed a ¾ of circle pattern, with only one small side of the forespore not covered by a red fluorescent signal (Fig. S3E, orange arrow). Importantly, in those sporangia presenting a ¾ of a circle localization of CotE-SNAP the MCD forespore pole was always covered by CotE-SNAP (Fig. S3E), in contrast to sporangia with a ¾ of a circle CotY-SNAP localization, which are mostly partly covered (Fig. S3B) or fully uncovered in the MCD region (Fig. S3A). This result show that the observation of a ¾ of a circle localization with a MCD forespore pole not encased by CotY-SNAP is linked to the observation of an off-center MCP cap.

Altogether, our results demonstrate that the encasement by CotE, and possibly CotY and ExsY, is a bidirectional mechanism, as it occurred through the localization of CotE at the MCD pole. Additionally, the last step of this encasement is asymmetric, as it covers only one longitudinal side of the forespore before the other one. However, in sporangia where the exosporium cap is normally separated from the OFM, a movement, around the forespore, of the proteins forming the MCP cap can hide this sequence of encasement. Strikingly, in the exosporium-less *B. subtilis*, the progression of CotE and CotZ localization is one single cap, two-unconnected caps and two-connected caps (11). Little is known about the transition between the two unconnected and connected caps, except that this localization of CotE depends on the transcriptional regulator SpoIIID (11). Here, thanks to our super-resolution images, we observed a similar pattern of CotE-SNAP localization in *B. cereus* cells defective in exosporium cap separation. Altogether, those observations support that the encasement mechanism by CotE, and CotE-controlled proteins is similar in *B. cereus* and *B. subtilis*.

**CotE is required for cap formation in *B. cereus.*** We found that, in the absence of *cotE,* CotY-SNAP failed to assemble as a cap (Fig. 2). As CotY is proposed to be a key component of the cap, we wondered whether the cap normally assembles in the absence of CotE, as in *B. anthracis* (8). We examined *cotE* sporulating cells by TEM and we never detected the presence of a cap in *cotE* sporangia in sporangia that had just completed the engulfment process (Fig. S4E) or in sporangia presenting a dehydrated core and signs of cortex formation (Fig. S4F). In contrast, a clearly separated cap structure was visible in WT sporangia at the same sporulation stages (Fig. S4B-C). In 80% of *cotE* sporangia with visible signs of coat deposition, as assessed using TEM, exosporium material formed large aggregates that appeared connected to the MCP forespore pole or located in the mother cell cytoplasm (Fig. S4G and H), while the exosporium was absent in 20% of the sporangia. Moreover, the accumulation of exosporium material observed with TEM overlapped with the fluorescence signal of ExsY-SNAP aggregates observed with SR-SIM in the mother cell cytoplasm (Fig. 2B, panel *f*). ExsY self-polymerization could lead to the accumulation of exosporium material seen in *cotE* sporangia, indicating that CotE is not required for ExsY polymerization (Fig. S4G-H).

An absence of cap in *B. anthracis* resulted in abnormal coat development (8). In line with this observation, 61% of *cotE* sporangia (as opposed to 9% in WT sporangia) exhibited an abnormal pattern of coat deposition, with coat material found at the MCP forespore pole, whereas other regions of the forespore were not covered by coat material (Fig. S4G). Together, these observations indicate a failure of cap assembly in *cotE* sporangia leading to an abnormal coat deposition and delayed exosporium formation in *B. cereus,* as in *B. anthracis cotE* mutant (8, 15). Finally, in line with these observations, no cap was observed in *cotE* sporangia imaged by SR-SIM (Fig. 2A-B, panels *d*). This also confirms that the SR-SIM signal detected at the time of formation of the interspace, just after engulfment completion, in WT sporangia, corresponds to the cap (Fig. 1, panels *d* and *e*, pink arrows).

**CotE and CotY form a complex at the cap independently of ExsY.** We show that CotE forms complexes with CotY and ExsY throughout sporulation (Fig. 4; see also the main text) and we wondered whether CotE and CotY formed a complex in the absence of ExsY. We thus performed a pull-down assay using the SNAP-capture matrix, on extracts prepared from sporulating cultures of an *exsY* mutant producing CotY-SNAP at 20°C. Note that the pathway of CotY-SNAP localization in *exsY* sporangia formed at 20°C or at 37°C were undistinguishable (Fig. 3A and S7B). We detected different forms of CotY-SNAP in cell extracts prepared from samples collected at hours 48 and 72 (Fig. S7A). CotY-SNAP was present in cells at hour 28 when imaged by fluorescence microscopy (Fig. S7B) but likely below our detection level by immunoblotting (Fig. S7A, extracts panel). CotE was detected as multimers from hour 28 and as monomers from hour 48 (Fig. S7A, extracts panel, two and one red asterisks, respectively). In the flow-through, after incubation of the extracts prepared from the *exsY* mutant producing CotY-SNAP from hour 48, we detected CotY-SNAP as monomers (one red asterisk), high molecular complex (two red asterisks), and less abundant fragments possibly resulting from proteolysis (blue asterisks). CotY-SNAP was pulled-down with the extracts produced from the *exsY* mutant producing CotY-SNAP at hour 48, but not at hour 72 (Fig. S7A, pull-down panel). We found that CotE was pulled-down by CotY-SNAP in extracts from the *exsY* mutant prepared at hour 48 (Fig. S7A, pull-down panel) while an unknown protein non-specifically recognized by the anti-CotE antibody was not and was lost in the flow-through (Fig. S7A, hour 0, green asterisk). This shows an association of CotE with CotY in the cap, independently of ExsY.

**SUPPLEMENTAL REFERENCES**

1. Todd SJ, Moir AJG, Johnson MJ, Moir A. 2003. Genes of *Bacillus cereus* and *Bacillus anthracis* encoding proteins of the exosporium. J Bacteriol 185:3373–3378.

2. Terry C, Jiang S, Radford DS, Wan Q, Tzokov S, Moir A, Bullough PA. 2017. Molecular tiling on the surface of a bacterial spore – the exosporium of the *Bacillus anthracis/cereus/thuringiensis* group. Mol Microbiol 104:539–552.

3. Krajčíková D, Lukáčová M, Müllerová D, Cutting SM, Barák I. 2009. Searching for Protein Protein Interactions within the *Bacillus subtilis* Spore Coat. J Bacteriol 191:3212–3219.

4. Jiang S, Wan Q, Krajcikova D, Tang J, Tzokov SB, Barak I, Bullough PA. 2015. Diverse supramolecular structures formed by self-assembling proteins of the *Bacillus subtilis* spore coat. Mol Microbiol 97:347–359.

5. Liu H, Qiao H, Krajcikova D, Zhang Z, Wang H, Barak I, Tang J. 2016. Physical interaction and assembly of *Bacillus subtilis* spore coat proteins CotE and CotZ studied by atomic force microscopy. J Struct Biol 195:245–251.

6. Krajčíková D, Forgáč V, Szabo A, Barák I. 2017. Exploring the interaction network of the *Bacillus subtilis* outer coat and crust proteins. Microbiol Res 204:72–80.

7. Liu H, Krajcikova D, Wang N, Zhang Z, Wang H, Barak I, Tang J. 2016. Forces and kinetics of the *Bacillus subtilis* spore coat proteins CotY and CotX binding to CotE inspected by single molecule force spectroscopy. J Phys Chem B 120:1041–1047.

8. Boone TJ, Mallozzi M, Nelson A, Thompson B, Khemmani M, Lehmann D, Dunkle A, Hoeprich P, Rasley A, Stewart G, Driks A. 2018. Coordinated assembly of the *Bacillus anthracis* coat and exosporium during bacterial spore outer layer formation. mBio 9:e01166-18

9. Ohye DF, Murrell WG. 1973. Exosporium and spore coat formation in *Bacillus cereus* T. J Bacteriol 115:1179–1190.

10. Wang KH, Isidro AL, Domingues L, Eskandarian HA, McKenney PT, Drew K, Grabowski P, Chua M-H, Barry SN, Guan M, Bonneau R, Henriques AO, Eichenberger P. 2009. The coat morphogenetic protein SpoVID is necessary for spore encasement in *Bacillus subtilis*. Mol Microbiol 74:634–649.

11. McKenney PT, Eichenberger P. 2012. Dynamics of spore coat morphogenesis in *Bacillus subtilis*. Mol Microbiol 83:245–260.

12. Steichen CT, Kearney JF, Turnbough CL. 2007. Non-uniform assembly of the *Bacillus anthracis* exosporium and a bottle cap model for spore germination and outgrowth. Mol Microbiol 64:359–367.

13. Boydston JA, Yue L, Kearney JF, Turnbough CL. 2006. The ExsY protein is required for complete formation of the exosporium of *Bacillus anthracis*. J Bacteriol 188:7440–7448.

14. Stewart GC. 2015. The exosporium layer of bacterial spores: a connection to the environment and the infected host. Microbiol Mol Biol Rev 79:437–457.

15. Giorno R, Bozue J, Cote C, Wenzel T, Moody K-S, Mallozzi M, Ryan M, Wang R, Zielke R, Maddock JR, Friedlander A, Welkos S, Driks A. 2007. Morphogenesis of the *Bacillus anthracis* Spore. J Bacteriol 189:691–705.
