# Supplementary material for: The Morphogenetic Protein CotE Positions Exosporium Proteins CotY and ExsY during Sporulation of Bacillus cereus
Source: mSphere. 2021 Apr 21;6(2):e00007-21. doi: 10.1128/mSphere.00007-21 (PMC8546674; doi:10.1128/mSphere.00007-21)
Supplement: TABLE S1 [file msphere.00007-21-st001.docx]

| Strains or plasmids | Name (designation in this work) | | Genotype/ phenotype | Source or reference | |  |
| --- | --- | --- | --- | --- | --- | --- |
| *B. cereus* | ATCC 14579 wo (WT) | | Cured from pclin15 plasmid | laboratory collection | |  |
|  | ATCC 14579 Δ*cotE (cotE)* | | *cotE*::*spc /* Spc^R^ | Bressuire et al 2016 | |  |
|  | ATCC 10876 (WT) | | *trp-1* /Str^R*^ | Johnson et al 2006 | |  |
|  | ATCC 10876 Δ*cotY (cotY)* | | *trp-1 cotY*::*erm* /Str^R^ Ery^R^ | Johnson et al 2006 | |  |
|  | ATCC 10876 Δ*exsY (exsY)* | | *trp-1* *exsY*::*spc /* Str^R^ Spc^R^ | “ | |  |
| *E. coli* | DH5α | | *fhuA2 lacU169 phoA glnV44 80= lacZM15 gyrA96 recA1 relA1 endA1 thi-1 hsdR17 L* | laboratory collection | |  |
|  | SCS110 | | *rpsL thr leu endA thi-1 lacY galK galT ara tonA tsx dam dcm supE44 (lac-proAB)* | “ | |  |
|  | BL21(DE3) | | F– *omp*T *hsdS_B_* (r_B_–, m_B_–) *gal dcm* (DE3) | Thermoscientifc | |  |
|  | Stellar | | F-, *endA1, supE44, thi-1, recA1, relA1, gyrA96, phoA, Φ80d lacZΔ M15, Δ(lacZYA-argF) U169, Δ(mrr-hsdRMS-mcrBC), ΔmcrA, λ-* | TakaraBio | |  |
| Plasmids | pDIA | | Spc^R^ | laboratory collection | |  |
|  | pFT47 | | Cm^R^ Tm^R^ | Pereira 2013 | |  |
|  | pHT304-18 | | Amp^r^ Ery^r^ | Sanchis et al 1996 | |  |
|  | pETDuet-1 | | Amp^R^ | Novagen | |  |
|  | pHT304-CotESNAP | | Amp^R^ Ery^R^ | this study | |  |
|  | pHT304-SNAPCotE | | Amp^R^ Ery^R^ | “ | |  |
|  | pHT304-pCotESNAP | | Amp^R^ Ery^R^ | “ | |  |
|  | pHT304-CotYSNAP | | Amp^R^ Ery^R^ | “ | |  |
|  | pHT304-CotYSNAPspc | | Amp^R^ Ery^R^::Spc^R^ | “ | |  |
|  | pHT304-ExsYSNAP | | Amp^R^ Ery^R^ | “ | |  |
|  | pETDuetCotEdeltaS | | Amp^R^ | “ | |  |
|  | pETDuetCotYHis | | Amp^R^ | “ | |  |
|  | pETDuetExsYHis | | Amp^R^ | “ | |  |
|  | pETDuetCotYHisCotEdeltaS | | Amp^R^ | “ | |  |
|  | pETDuetExsYHisCotEdeltaS | | Amp^R^ | “ | |  |
| Primer | | | Sequence | | |  |

| SNAP-RC-IF2-FW | ATGCCTGCAGGTCGACTTTCCTTACCCAAGTCCT |
| --- | --- |
| SNAP-RC-IF2-RV | AATGTCCGAATTTAGCTCGAGGGAGGAACTACT |
| pcotERC-IF2-FW | CTAAATTCGGACATTCGTAAC |
| pcotERC-IF2-RV | CAGTGCCAGTGAATTCGATGTACTAAAGGGTGAAA |
| SpcpHTCotYStyI-Fw | TACAAGCGTACCTTGGCGATCTTTTGTTTATAAGTG |
| SpcpHTCotYSnabI-Rv | TTTGAAACAAAGTACGTAGATCTGTATAATAAAGAATAATT |
| pETDuetExsYHis(2)-Fw | TGGTGGTGGGGATCCGAGTTGTAACGAAAATAAACACC |
| pETDuetExsYHis-Rv | TGGTGGTGGAAGCTTTTAGATAGTAACGTCGCGTAAGC |
| pETDuetCotYHis(2)-Fw | TGGTGGTGGGGATCCGAGCTGCAATTGTAACGAAGACC |
| pETDuetCotYHis-Rv | TGGTGGTGGAAGCTTTTAAATAGAAACATCGCGTAAGC |
| pETDuetcotEFw | TGGTGGTGGAGATCTATGTCCGAATTTAGAGAGA |
| pETDuetcotERv | TGGTGGTGGCTCGAGTTACTCTTCTTCTGCATCAACG |

*R, resistant to: streptomycin (Str), spectinomycin (Spc), erythromycin (Ery), ampicillin (Amp)
