# Supplementary material for: The Morphogenetic Protein CotE Positions Exosporium Proteins CotY and ExsY during Sporulation of Bacillus cereus
Source: mSphere. 2021 Apr 21;6(2):e00007-21. doi: 10.1128/mSphere.00007-21 (PMC8546674; doi:10.1128/mSphere.00007-21)
Supplement: TEXT S2 [file msphere.00007-21-s0002.docx]

**SUPPLEMENTAL MATERIAL AND METHODS**

**The morphogenetic protein CotE positions exosporium proteins CotY and ExsY during sporulation of *Bacillus cereus***

Armand Lablaine^a^, Monica Serrano^b^, Christelle Bressuire-Isoarda, Stephanie Chamot^a^, Isabelle Bornard^c^, Frederic Carlin^a^, Adriano O. Henriques ^b*^ and Veronique Broussolle ^a*^

^a^ INRAE, Avignon Université, UMR SQPOV, F-84000 Avignon, France

^b^ Instituto de Tecnologia Quimica e Biologica, Universidade Nova de Lisboa, 2780-157 Oeiras, Portugal

^c^ INRAE, Pathologie végétale, F-84143 Montfavet, France

* address correspondence to Veronique Broussolle, [veronique.broussolle@inrae.fr](mailto:veronique.broussolle@inrae.fr) and Adriano O. Henriques, aoh@itqb.unl.pt

Running title: Assembly of *Bacillus cereus* exosporium

Keywords: Spore, morphogenetic proteins, exosporium, SR-SIM

**SUPPLEMENTAL MATERIAL AND METHODS**

**Construction of SNAP fusions and expression plasmids.** The *cotY-SNAP* and *exsY-SNAP* fusions were synthesized by ATUM ([www.atum.bio](http://www.atum.bio/), Newark, CA). Briefly, synthetic inserts corresponding to: i) the *cotY* gene (*bc_1222*) and the 264 bp sequence upstream of the *cotY* start codon or; ii) *exsY* gene (*bc_1218*) and the 265 bp sequence upstream of the *exsY* start codon of *B. cereus* ATCC14579, followed by a GCAGCTGCT linker and the SNAP sequence (1) were inserted into the pHT304-18 plasmid (2), using *Sal*I and *EcoR*I restriction sites, giving rise to the pHT304-CotYSNAP and pHT304-ExsYSNAP plasmids, respectively (Table S1). To allow introduction of the CotY-SNAP-producing plasmid into a *cotY-* strain (Erm^r^), the spectinomycin (Spc) resistance was amplified from the pDIA plasmid with SpcpHTCotYStyI-Fw and SpcpHTCotYSnabI-Rv primers (Eurogentec) (Table S1), containing a 15bp-extension homologous to the ends of a *Sty*I/*SnaB*I digested pHT304-CotYSNAP. The fragment was then introduced into pHT304-CotYSNAP using the In-fusion cloning (Takarabio), interrupting the Erm resistance gene and yielding pHT304-CotYSNAPspc. The transcriptional fusion pcotESNAP was obtained by cloning the SNAP tag sequence, amplified from pFT47 using primers SNAP-RC-IF2-FW and SNAP-RC-IF2-RV, under the control of the 189bp fragment preceding the start codon of the *cotE* open reading frame (*bc_3770*), amplified from *B. cereus* ATCC14579 genomic DNA with primers pcotERC-IF2-FW and pcotERC-IF2-RV (Table. S2). These primers contained a 15bp -extension for In-fusion cloning of the two fragments into pHT304-18 digested with *Sal*I and *EcoR*I. All plasmids were first introduced into *E. coli* DH5α and clones were confirmed by PCR and/or DNA sequencing. Plasmids were then transferred to *E. coli* SCS110; the resulting unmethylated plasmids were then transferred to the *B. cereus* strains by electroporation, as described (3). The pETDuet-1 vector (Novagen) was used for co-expression of different combinations of proteins and pull-down assays. The *cotY*, *exsY* and *cotE* genes were amplified from *B. cereus* ATCC14579 genomic DNA and introduced into pETDuet-1 to generate His tag fusions. The His-tag sequence was fused to the 5´-end of *cotY* or *exsY* primers usingpETDuetCotY.His(2)-Fw, pETDuetCotYHis-Rv, pETDuetExsY.His(2)-Fw and pETDuetExsYHis-Rv, carrying *BamH*I and *Hind*III restriction sites. After digestion, the amplified fragments were ligated into *BamH*I and *Hind*III digested pETduet-1, to yield the pETDuetCotYHis and pETDuetExsYHis plasmids (Table 1). In the same way, *cotE* gene was amplified using primers pETDuetcotEFw and pETDuetcotERv, carrying *Bgl*II and *Xho*I sites and inserted in these two plasmids and in pETduet-1, resulting in plasmids pETDuetCotYHisCotEdeltaS, pETDuetExsYHisCotEdeltaS and pETDuetCotEdeltaS, respectively. These plasmids were then introduced in *E. coli* Stellar competent cells (Takarabio) and then into BL21 (DE3) for protein production.

**Sporangia and spore lysis.** Sporulating cells or purified spores from cultures in SMB or from FNA plates were lysed by bead beating using a Fastprep II (MP Biomedical) for 6 cycles at speed 5 in buffer A [100mM NaCl, 10mM Tris-HCl (pH8.0), 10% glycerol, 1mM DTT]. Lysates were cleared by centrifugation for 10 min at 7,000 x g. Protein concentration was determined using a Bradford assay.

**SNAP pull-down assays.** For SNAP pull-down assays, 160µL of a 50% slurry of SNAP-Capture resin (NEB) were equilibrated with buffer A (see above), as recommended by the manufacturer. Cleared lysates from whole cell extracts (Extracts in Fig. 4, S6 and S7A) prepared from sporulating cultures from hours 20 to 72 (0.2 mg.ml^-1^) or from purified spores (0.15 mg.ml-1), were immobilized in 160µL of a shaken suspension of beads equilibrated in buffer A for 1 h at 4°C and then for 1 h at room temperature. After incubation, the mix was centrifuged at 11,000 x g for 1 min at 4°C and the supernatant was kept for analysis (flow-through in Fig. 4, S6 and S7). The beads were washed three times with 1mL of buffer B [200mM NaCl, 10mM Tris-HCl (pH8), 10% glycerol, 1mM DTT] agitated for 1 min and centrifuged at 11,000 x g for 1 min at 4°C. Beads were then resuspended in 50µL of buffer B, heated at 100°C for 5 min and kept at -20°C (Pull-down in Fig. 4, S6 and S7). Experiments were performed at least twice, giving the same results.

**Protein co-production in *E. coli* and pull-down assays.** *E. coli* BL21 (DE3) containing the various plasmids (above) were grown at 37°C in LB with 100 µg.mL^-1^ ampicillin. Protein co-production was induced in cultures at an OD_600nm_ of 0.6, by addition of 1mM isopropyl-β-galactoside (IPTG) for 3h at 37°C. Cells were then collected by centrifugation and stored at -20°C until analysis. A 4mL volume of the cell suspension was lysed in 1mL of urea buffer [8M urea, 150mM NaCl, 25mM TrisHCl (pH 8.0), 2% SDS]­ using a Fastprep (5 cycles of 45s at speed 6.5) and boiled for 20 min at 90°C. After sedimentation, 250 µL of supernatant were mixed with equilibration buffer (PBS, 10 mM imidazole) and the mix was incubated for 30 min at room temperature, with 100 µL of Ni^2+^ beads, equilibrated as recommended by the manufacturer. Then, equilibrated beads were washed three times with washing buffer (PBS with 25mM imidazole) and proteins were eluted once with 500 mM imidazole 8M urea and then twice with 1M imidazole 8M urea. Experiments were performed twice, giving the same results.

**Immunoblotting analysis.** Proteins (5 μg) of the pull-down elutions and flow-through (10µL) were mixed with a 5X buffer [300mM Tris (pH 6.8), 50% glycerol, 10% SDS, 5% β-mercaptoethanol, bromophenol blue], heated 5 min at 100°C and resolved on 12% SDS-PAGE gels, which were transferred to nitrocellulose membranes using the Iblot II system (Invitrogen). Protein transfer onto the membrane was confirmed by Red Ponceau staining. Membranes were incubated for 45 min with blocking buffer [8% (wt/vol) non-fat milk in PBS], washed three times with PBST (PBS containing 0.1% Tween 20) for 10 min and incubated with the appropriate antibodies for 2 h at room temperature. For pull-down SNAP assays, anti-*B. cereus* CotE and anti-SNAP polyclonal antibodies (diluted at 1:500 and 1:1,000 in blocking buffer, respectively) were used. For immunoblotting analysis of co-expression pull-down, we used an anti-*B. anthracis* CotE polyclonal antibody (at dilution 1:25,000) (4), and a monoclonal anti-His peroxidase-conjugated antibody (at 1:50,000). Membranes were washed three times with PBST and then incubated with secondary anti-rabbit peroxidase-conjugated antibodies (Sigma Aldrich, at 1:10,000) for 2 h. Membranes were washed three times with PBST and incubated with ECL peroxidase substrate (Thermofischer).

**SUPPLEMENTAL REFERENCES**

1. Pereira FC, Saujet L, Tomé AR, Serrano M, Monot M, Couture-Tosi E, Martin-Verstraete I, Dupuy B, Henriques AO. 2013. The Spore Differentiation Pathway in the Enteric Pathogen *Clostridium difficile*. PLOS Genet 9:e1003782.

2. Sanchis V, Agaisse H, Chaufaux J, Lereclus D. 1996. Construction of new insecticidal *Bacillus thuringiensis* recombinant strains by using the sporulation non-dependent expression system of cryIIIA and a site specific recombination vector. J Biotechnol 48:81–96.

3. Brillard J, Susanna K, Michaud C, Dargaignaratz C, Gohar M, Nielsen-Leroux C, Ramarao N, Kolstø A-B, Nguyen-the C, Lereclus D, Broussolle V. 2008. The YvfTU Two-component System is involved in plcR expression in *Bacillus cereus*. BMC Microbiol 8:183.

4. Giorno R, Bozue J, Cote C, Wenzel T, Moody K-S, Mallozzi M, Ryan M, Wang R, Zielke R, Maddock JR, Friedlander A, Welkos S, Driks A. 2007. Morphogenesis of the *Bacillus anthracis* Spore. J Bacteriol 189:691–705.
